# The *de novo *sequence origin of two long non-coding genes from an inter-genic region

**DOI:** 10.1186/1471-2164-14-S8-S6

**Published:** 2013-12-09

**Authors:** Yulin Dai, Shengdi Li, Xiao Dong, Han Sun, Chao Li, Zhi Liu, Beili Ying, Guohui Ding, Yixue Li

**Affiliations:** 1 Key Laboratory of Systems Biology, Shanghai Institutes for Biological Sciences, Chinese Academy of Sciences, 320 Yueyang Rd. Shanghai 200031, PR China; 2 Graduate School of Chinese Academy of Sciences, 19 Yuquan Rd. Beijing 100049, PR China; 3 Shanghai Center for Bioinformation Technology, 1278 Keyuan Rd. Shanghai 201203, PR China; 4 School of Life Sciences, Fudan University, 220 Handan Rd. Shanghai 200433, PR China

**Keywords:** Overlapping transcripts, Sequence Origin of *Pldi *and *Ak158810 *loci, Conserved Element, Substitution rate

## Abstract

**Background:**

The gene Polymorphic derived intron-containing, known as *Pldi*, is a long non-coding RNA (lncRNA) first discovered in mouse. Although parts of its sequence were reported to be conserved in rat and human, it can only be expressed in mouse testis with a mouse-specific transcription start site. The consensus sequence of *Pldi *is also part of an antisense transcript *AK158810 *expressed in a wide range of mouse tissues.

**Result:**

We focused on sequence origin of *Pldi *and *Ak158810*. We demonstrated that their sequence was originated from an inter-genic region and is only presented in mammalians. Transposable events and chromosome rearrangements were involved in the evolution of ancestral sequence. Moreover, we discovered high conservation in part of this region was correlated with chromosome rearrangements, CpG demethylation and transcriptional factor binding motif. These results demonstrated that multiple factors contributed to the sequence origin of *Pldi*.

**Conclusions:**

We comprehensively analyzed the sequence origin of *Pldi*-*Ak158810 *loci. We provided various factors, including rearrangement, transposable elements, contributed to the formation of the sequence.

## Introduction

Although pervasively transcribed, only 5%-10% of the human genome is covered by mRNA and spliced non-coding RNAs, and the majority of which does not encode proteins [1]. Long non-coding RNAs (lncRNAs) are defined as transcribed non-coding RNA larger than 200 nt in length, which plays an essential role in regulating gene expression, chromatin functions [2]. As lncRNAs act as biological building blocks, it is necessary to understand the process of developing new lncRNA genes [3].

The emergence of a functional lncRNA gene could be summarized into various evolutionary scenarios, including metamorphosis of a protein coding gene, derived from a genomic region previously devoid of exonic sequence, duplication by retro-transposition, and emergence following tandem duplication or insertion of transposable elements [1,4]. For most of the scenarios, comprehensive studies have been established on specific lncRNA genes with well-known functions, such as *XIST*, *HOTAIR *[5,6]. However, little was known about developing a new lncRNA gene from a non transcribed genomic region. The mechanism of the de novo origin of a lncRNA gene remains to be clarified.

Previous study on *de novo *protein has accounted for that those seemingly dispensable sequences in non-genic regions could generate adaptive functional proteins through evolution. The *de novo *birth and development of a potential protein coding gene is in line with increasing open reading frame (ORF) length and conservation through the natural selection benefited from random translation on genome [3,7,8]. Like proteins, the occasional transcription and changing events in non-genic sequences could provide raw material generating *de novo *lncRNAs [9]. Here, we focused on the sequence origin of a lncRNA in an intergenic region, demonstrating its sequence components and changes within species.

*Pldi *gene was previously identified and defined as an intergenic originated lncRNA gene, which is overlapped with a putative opposite-strand transcript, *AK158810 *(Additional File 1). *Pldi *locates within a 200 kb region that is free of annotated transcripts or expressed sequence tags (ESTs) in rat and humans, which raise the possibility of de novo emergence of the *Pldi*-*AK158810 *loci (about 20 kbps-long). Knocking out *Pldi *would reduce sperm motility and testis weight, indicating that *Pldi *has the ability in regulating the expression levels of other genes in testis [10]. Numerous functional non-coding RNAs have been demonstrated to regulate gene expressions through an antisense mechanism, playing an important role of gene overlapping in non-coding RNA functions [11-13]. On the contrary, few studies discussed the origin of overlapping non-coding RNAs due to lacking of clear markers, like ORF in protein.

In this study, we conducted a comprehensive analysis on the sequence origin of mouse *Pldi*-*Ak158810 *loci. We evaluated various factors that contribute to the origin, and gave adequate evidence to prove the de novo origin of this loci. Moreover, we found that *Pldi*-*Ak158810 *established its fixation from a specific overlapping region some time before emergence. We further discussed the potential role of the local element in the evolution and fixation of this orphan lncRNA gene loci.

## Materials and methods

### Genomes and sequences

The 13 genomes of vertebrates used in this study were downloaded from UCSC genome database http://hgdownload.soe.ucsc.edu/downloads.html. Genome versions of these 13 genomes are in Additional File 2. The sequence of *Pldi*-*Ak158810 *loci was picked from mouse (GRCm 38) export data in Ensembl http://www.ensembl.org.

### ORF analysis

Sequences of EST *Ak158810 *were checked to find all the potential open reading frames, by using ORF finder (Open Reading Frame Finder) by default minimum frame size. The ORF finder is accessible in this website server http://www.ncbi.nlm.nih.gov/gorf/gorf.html[14]

### Sequence comparison and alignment, phylogeny analysis

We used nucleotide Blast (Basic Local Alignment Search Tool) to detect homology between *Pldi-Ak158810 *nucleotide sequence and vertebrate genomes, a cutoff for identity was set at 80%. Protein Blast was used to find protein coding genes homologue to the genes flanking with *Pldi *and *Ak15880*. ClustalW http://www.clustal.org/download/current/ was used to align protein and nucleotide sequences [15]. MEGA5.1 was used to construct neighbor-joining phylogenic tree [16]. The genomic alignment of 30 vertebrates by MultiZ was downloaded from UCSC [17,18]. All genomes were mapped to the mouse chromosomes.

### Repeats and transposable elements annotation

Repeats and transposable elements were annotated by Repeatmasker program. Sequences of *Pldi*-*Ak158810 *were submitted to the Repeatmasker website http://www.repeatmasker.org version 4.0.1, which uses default parameters. The repeat class were transformed and grouped as SINE, LINE, DNA, LTR and others. In the analysis of ancient transposable elements, we did not include simple repeats and low complexity sequences [19].

### Model for substitution rate change

**Figure 1:**
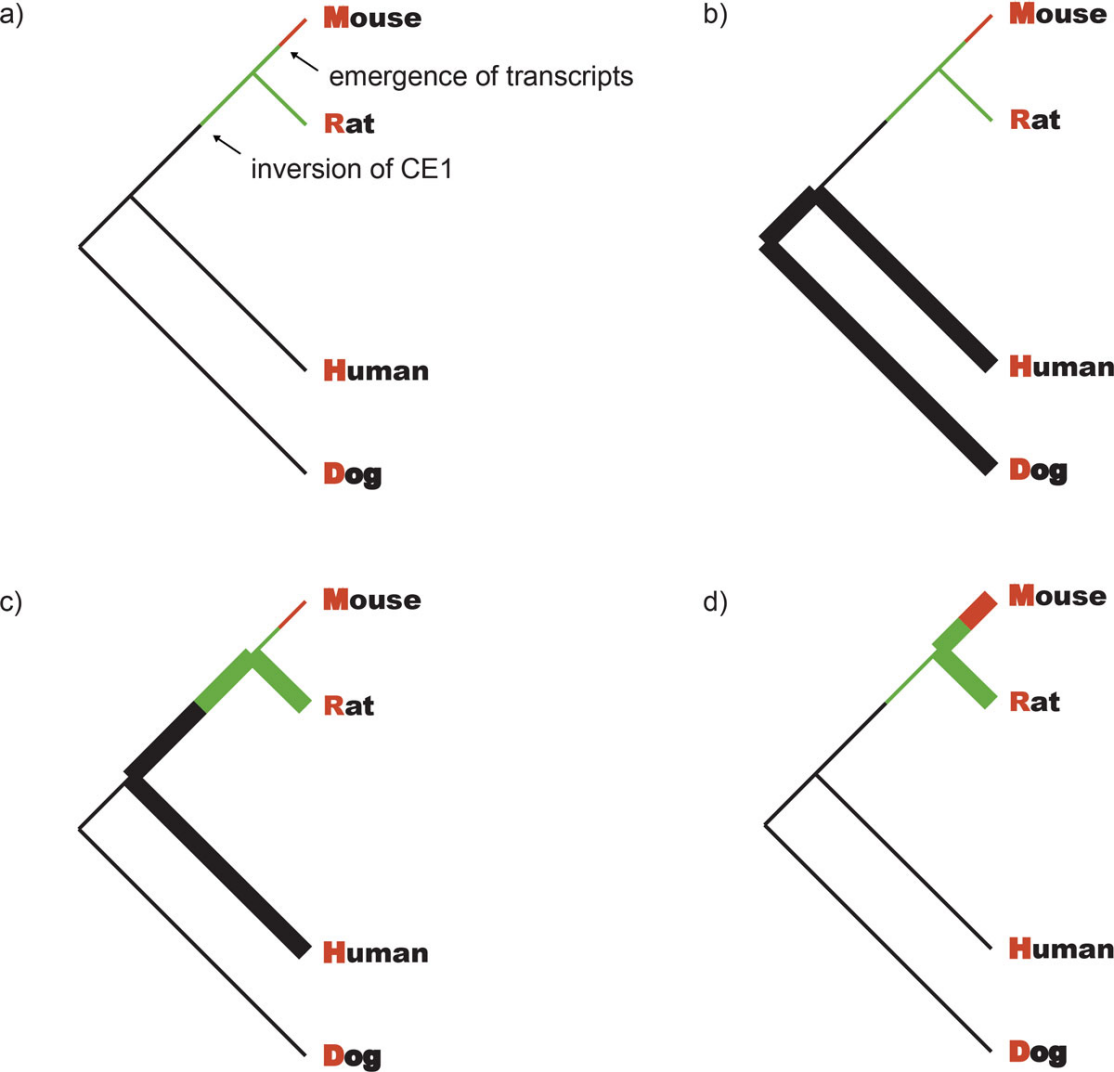
**Assumptions of the model**. We used surrounding gene exons as reference. We assumed the substitution rate relative to those exons would not change too much if no selection pressures affect this region. For test sequence, we defined three constant substitution rates on the species tree, to test the rate change at two time point: inversion and emergence of *Pldi-Ak158810 *transcription. (a) Red lines represent for substitutions after gene birth, at rate r_2_; green lines represent for substitutions after two observed inversion, and before gene birth, at rate r_1_; black lines represent for substitutions before inversion, at rate r_0_. We were concerned about the rate change relative to surrounding gene exons, all the rates were normalized by the variable reference rate r_R_. (b-d) r_averXY _were calculated by dividing test sequence XY distance by reference XY distance, which indicates a relative substitution rate on the bold path; three r_averXY _will be similar if substitution rates do not vary significantly in three stages.

A simple model was constructed to test the substitution rate change relative to exons of surrounding genes. Three constant substitution rates were defined as: r_0_, rate before inversion; r_1_, rate after inversion and before gene birth; r_2_, rate after gene birth. As surrounding genes' reference sequence, the rates were summarized as r_R _encompassing the whole phylogenetic tree. We assumed the substitution rates relative to those exons will not change significantly if no selection pressures affect this region. A time interval was estimated instead of an exact time point, of the inversion and gene birth, so we used two variables k_1_, k_2_, ranging from 0~1 to reflect the timing of two events. k_1 _is the proportion of the time from present to the common ancestor of human and rat, in which the inversion has occurred. k_2 _is the proportion from present to the common ancestor of mouse and rat, for the emergence of lncRNA transcription. The distance between species X and Y d_XY _can be approximately calculated as d_XY _= 2·r_XY_·t_XY _, if the rate is regarded as constant. Under the assumptions of our model (Figure 1), for test sequence we get ():

(1) $${{\text{d}}^{\text{test}}}_{\text{HD}}=\text{2}\cdot {\text{r}}_{0}\cdot {\text{t}}_{\text{HD}},$$

(2) $${{\text{d}}^{\text{test}}}_{\text{HR}}=\left(\text{1}+{\text{k}}_{\text{1}}\right)\cdot {\text{r}}_{0}\cdot {\text{t}}_{\text{HR}}+\left(\text{1}-{\text{k}}_{\text{1}}\right)\cdot {\text{r}}_{\text{1}}\cdot {\text{t}}_{\text{HR}},$$

(3) $${{\text{d}}^{\text{test}}}_{\text{MR}}=\left(\text{1}+{\text{k}}_{\text{2}}\right)\cdot {\text{r}}_{\text{1}}\cdot {\text{t}}_{\text{MR}}+\left(\text{1}-{\text{k}}_{\text{2}}\right)\cdot {\text{r}}_{\text{2}}\cdot {\text{t}}_{\text{MR}},$$

where d^test^_HD_, d^test^_HR_, d^test^_MR _are the genetic distance of the test sequences between human and dog, human and rat, mouse and rat, respectively, t_HD_, t_HR_, t_MR _represent for the divergent time between each pair of species.

For reference sequence,

(4) $${{\text{d}}^{\text{Ref}}}_{\text{HD}}=\text{2}\cdot {\text{r}}_{\text{R}}\cdot {\text{t}}_{\text{HD}},$$

(5) $${{\text{d}}^{\text{Ref}}}_{\text{HR}}=\text{2}\cdot {\text{r}}_{\text{R}}\cdot {\text{t}}_{\text{HR}},$$

(6) $${{\text{d}}^{\text{Ref}}}_{\text{MR}}=\text{2}\cdot {\text{r}}_{\text{R}}\cdot {\text{t}}_{\text{MR}},$$

where d^Ref^_HD_, d^Ref^_HR_, and d^Ref^_MR _are the genetic distance of the reference exon sequences between human and dog, human and rat, mouse and rat.

We calculated three average substitution rates,

(7) $${\text{r}}_{\text{averHD}}=\;{{\text{d}}^{\text{test}}}_{\text{HD}}/\;{{\text{d}}^{\text{Ref}}}_{\text{HD}}=\;{\text{r}}_{0}/\;{\text{r}}_{\text{R}},$$

(8) $${\text{r}}_{\text{averHR}}=\;{{\text{d}}^{\text{test}}}_{\text{HR}}/\;{{\text{d}}^{\text{Ref}}}_{\text{HR}}=\left[\left(\text{1}+{\text{k}}_{\text{1}}\right)\cdot {\text{r}}_{0}+\left(\text{1}-{\text{k}}_{\text{1}}\right)\cdot {\text{r}}_{\text{1}}\right]/\;{\text{r}}_{\text{R}},$$

(9) $${\text{r}}_{\text{averMR}}=\;{{\text{d}}^{\text{test}}}_{\text{MR}}/\;{{\text{d}}^{\text{Ref}}}_{\text{MR}}=\left[\left(\text{1}+{\text{k}}_{\text{2}}\right)\cdot {\text{r}}_{\text{1}}+\left(\text{1}-{\text{k}}_{\text{2}}\right)\cdot {\text{r}}_{\text{2}}\right]/\;{\text{r}}_{\text{R}},$$

where 0 < k_1_, k_2 _<1. r_averHD_, r_averHR _and r_averMR _are the average substitution rates at different stages. If r_averHR _<(>) r_averHR _, we get r_0_<(>) r_1_. Similarly, a reduced r_2 _will produce a lower r_averMR_, as the only envolving path affected by r_2_.

We used ClustalW to realign the conserved elements in *Pldi*-*Ak158810 *and exons of surrounding four genes, manually remove sites with low similarity by Bioedit http://www.mbio.ncsu.edu/bioedit/bioedit.html. All the four genes were merged into one single alignment. Then a Maximum Likelihood (ML) tree and distance matrix was estimated by PAML 4.6 baseml for each alignment) [20].

### Methylations data

We collected two sources of methylation data as a comparison, one is from mouse tissue, the other is from human ENCODE data.

Mouse brain methylation data was obtained from forebrain tissue of lab mouse (GSM809309)

The probability of methylation was estimated with both methylated and unmethylated fragment information (Additional File 3) [21].

Demethylation data from human UCSF brain methylation database viewed with UCSC genome browser was implemented to detect the DNA methylation in the human homologue region of *Pldi*-*Ak158810 *loci, which was displaced in Additional file 3[22].

### RNA-seq data

RNA-seq data is from Encode Cold Spring Harbor Lab (CSHL) RNA-seq, and there are 5 types of tissues included (heart, kidney, ovary, spleen and testis). We viewed this data using UCSC genome browser [23].

### Transcriptional factor binding site data

Human, Mouse, Rat (HMR) Conserved Transcriptional Factor Binding Site (TFBS) was implemented to displace the potential binding sites of these two highly conserved regions [24].

http://www.biobase-international.com/library/transfac

## Results

### The co-emergence of *Pldi *and *Ak158810 *transcription

We studied the emergence time of *Pldi *and Ak158810. *Pldi *locates in an inter-genic region free of any human and rat EST signals, indicating that *Pldi *and its antisense putative gene generating *Ak158810 *were not transcribed before the divergence of rodents. In mouse lineages, RNA transcript of *Pldi *has been discovered [10]. To validate the transcript *Ak158810*, we compared its 2.9 kb sequence with mouse EST database from NCBI. The result confirmed the transcription of *Pldi *antisense strand, and EST hits matched with splicing of the first and second exons of *Ak158810 *(Additional File 4). We further analyzed the open reading frames (ORFs) in *Ak158810 *RNA, The longest ORF is shorter than 110 amino acids. (Two AUG codons with shorter reading frames ~70 amino acids preceded this long ORF) (Additional File 5). It indicates that *Ak158810 *is not likely to encode proteins. Our results, along with previous knowledge [10], showed that the *Pldi *and *Ak158810 *are two mouse-specific lncRNAs located on anti-sense strand to each other. These evidences suggest that *Pldi*, and its putative antisense lncRNA, *Ak158810*, were first transcribed at similar time between the divergence of mouse and rat.

### *Pldi-Ak158810 *loci is conserved in mammals and originated from an intergenic region

**Figure 2:**
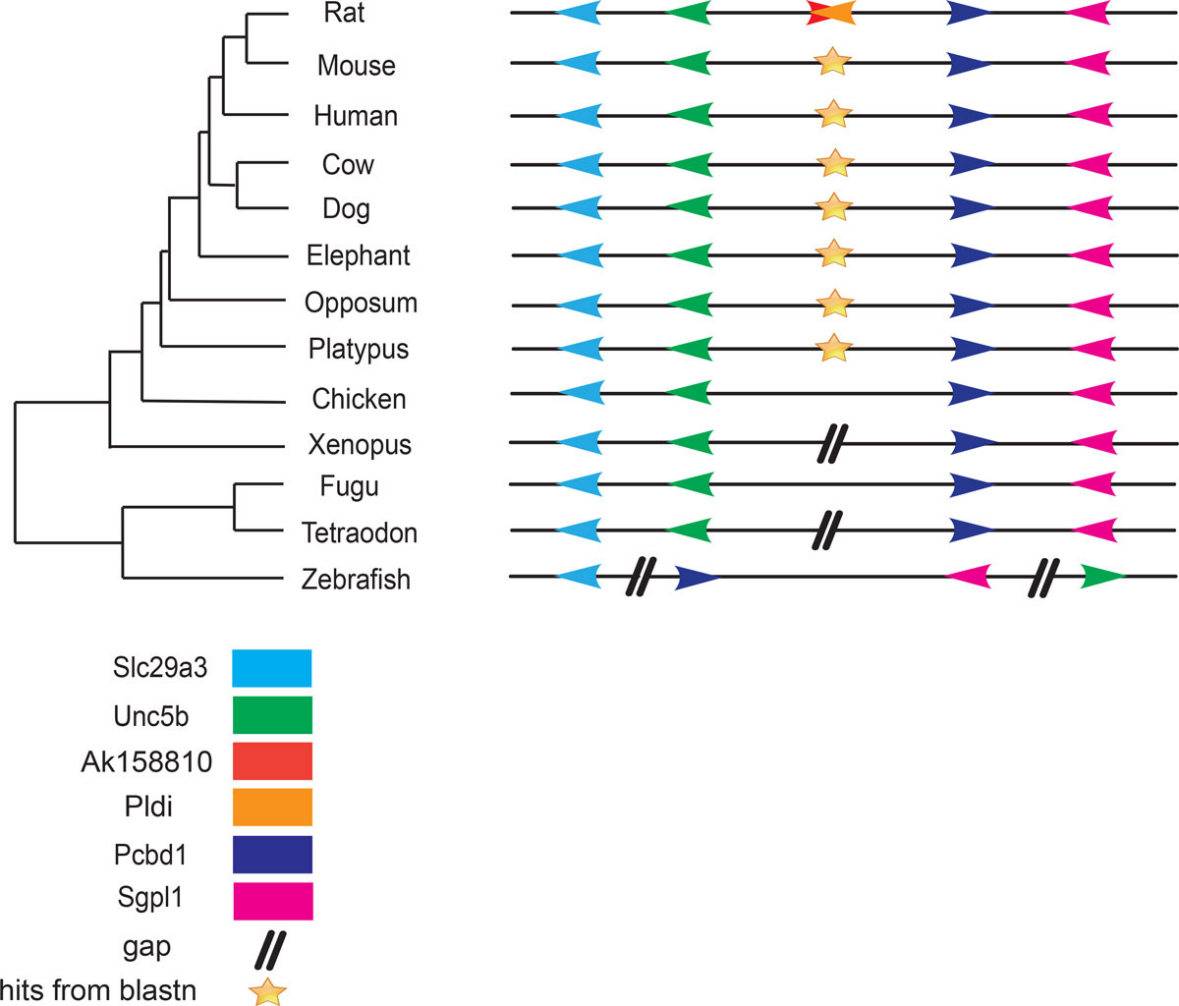
**Phylogenetic distribution of *Pldi-Ak158810 *loci and its surrounding genes within vertebrate species**. The phylogenetic tree of 13 vertebrates was adapted from a widely accepted tree topology [5,32]. The branch length does not represent the distance between each species and no molecular clock model was assumed. Different gene highlighted with marks of different colors. We could find the hits from Blastn in all mammals, whereas no hits in non-mammalian species. We could observe that all these 4 flanking protein are ordered laid around the *Pldi-Ak158810 *loci. In contrast, in non-mammalian species, some big gaps (larger than the average distance ~200 kb in mammals) inserted into the 4 flanking protein region, which made the order of the 4 proteins changed. The gap in Xenopus might due to the incomplete genomic description. Result of Blastn could be found in Additional File 6.

To study the evolution of *Pldi-Ak158810 *loci, we searched for homologues of *Pldi *and *Ak158810 *loci in 13 vertebrates. First, homologs of *Pldi*-*Ak158810 *sequence were found in all mammals by using Blastn. Except a transposable element in rat and mouse, all the homologs are between the region of unc5b and pcbd1 in mammalian classes. It demonstrates that parts of *Pldi-Ak158810 *loci were already present in the mammalian cen-ancestor. Meanwhile, we failed to detect significant sequence similarity to *Pldi-Ak158810 *loci in non-mammalian vertebrates with Blastn (Figure 2 & Additional File 6).

**Figure 3:**
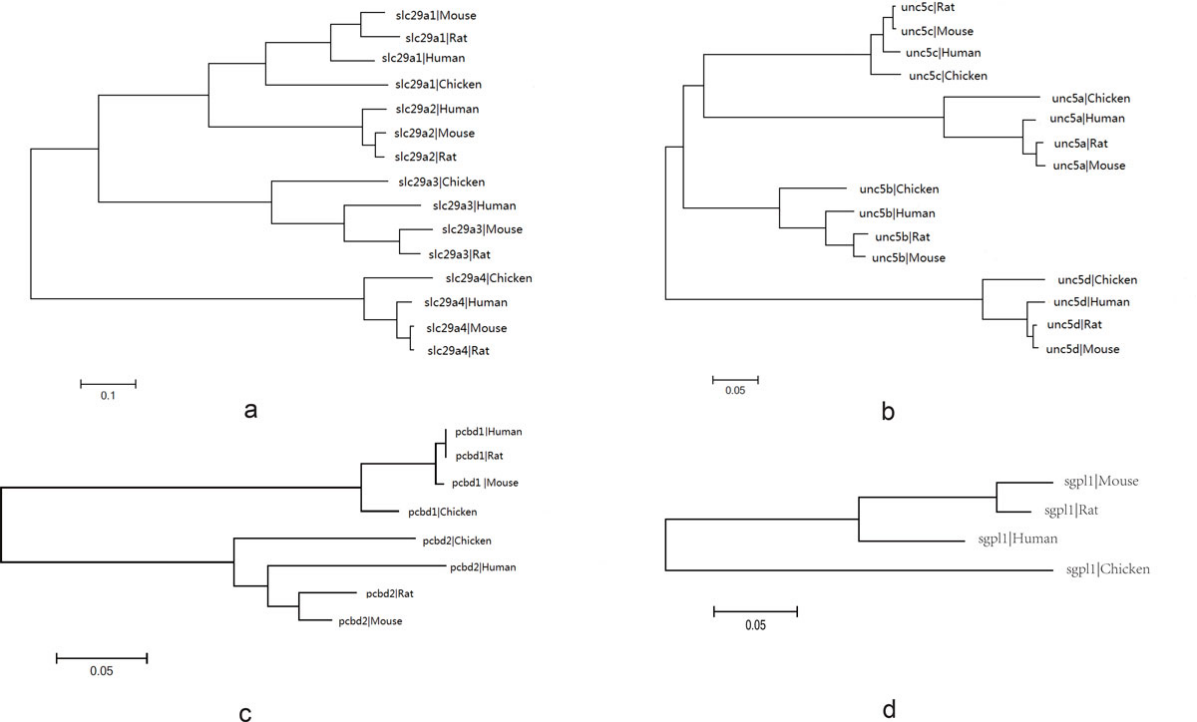
**Clustering paralogues and orthologues of 4 flanking proteins**. We used clustering to identify the paralogues and orthologues of the 4 flanking proteins (a: *slc29a3*, b: *unc5b*, c: *pcbd1*, d: *sgpl1*) in 4 species (mouse, rat, human and chicken). The result beyond indicates these *Pldi-Ak158810 *loci surrounding regions linkaged in these 4 species and these 4 flanking genes have been free from duplication of their paralogues since the time of their cen-ancestor. *Sgpl1 *doesn't contain a paralogue and it has the same topological structure as the other genes.

In mouse, a ~450 kb genomic region surrounding *Pldi *and *Ak158810*, contains four protein coding genes (slc29a3, unc5b, pcbd1 and sgpl1) that have orthologous genes in vertebrates (Figure 2 & Figure 3). In 12 vertebrates, the gene content, order and orientation of four flanking genes are perfectly conserved. In zebrafish, the linkage of the four genes is broken (Figure 2). However, this does not correspond to the ancestral state of non-tetrapod vertebrates, for it is the only mismatch among three kinds of fish (fugu, tetraodon, zebrafish). These results denoted that the genomic region where *Pldi *and *Ak158810 *emerged is stable since the cen-ancester of vertebrates, which indicated that *Pldi-Ak158810 *loci originated from an intergenic region linkaged in non-tetrapod vertebrates and remained conservation in mammals

### The origins of exons and introns

**Figure 4:**
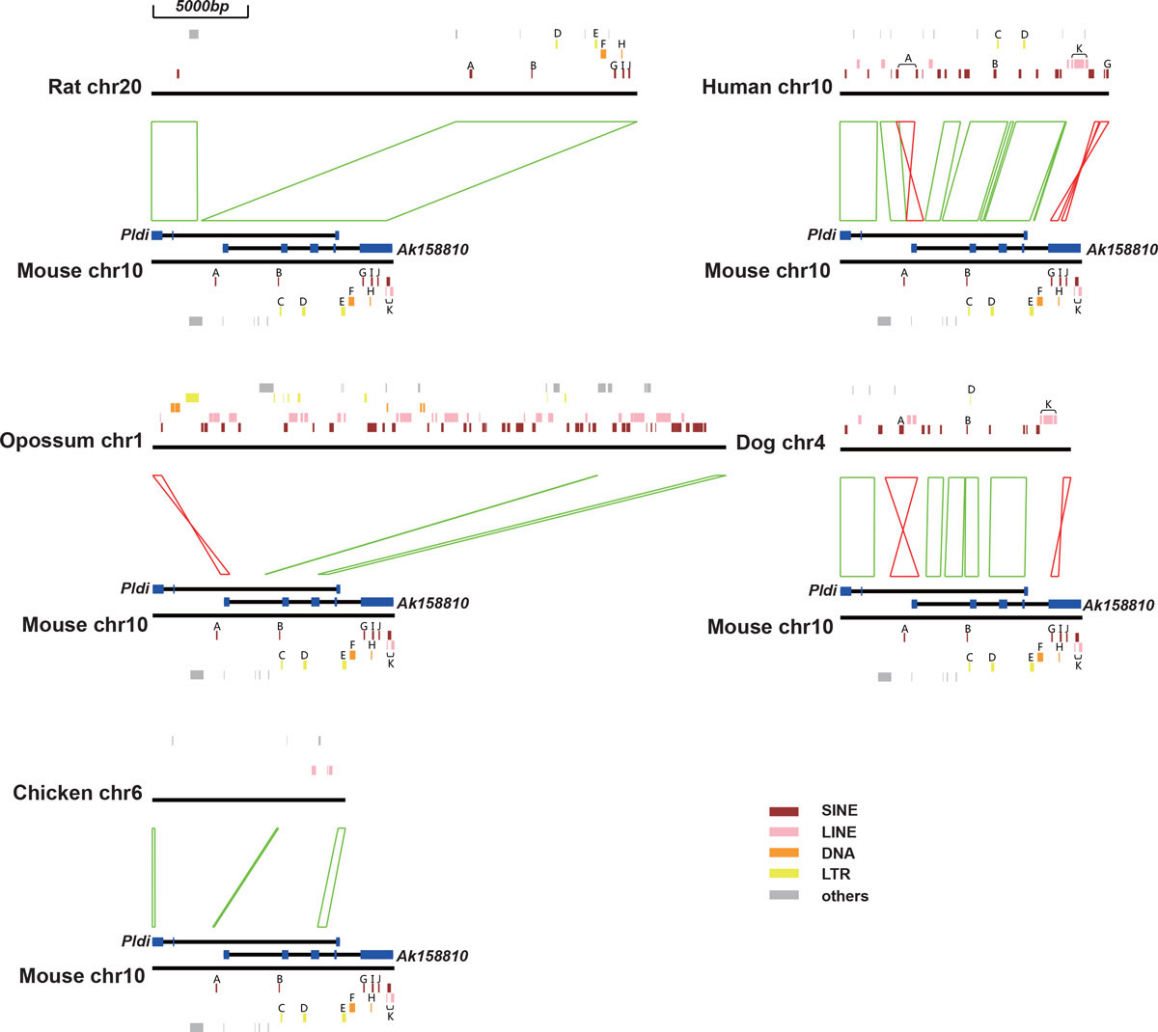
**Alignment of mouse and other five species (rat, human, dog, opossum, chicken): Positions of transposable elements at *Pldi-Ak158810 *loci**. The linkages between two chromosome fragments mean one or a few adjacent blocks in the alignment, and red ones mean inversion. Various class of transposable elements are plot in five colors. SINE, short interspersed nuclear elements; LINE, long interspersed nuclear elements; DNA, DNA repeat elements; LTR, long terminal repeat elements, including retroposons; Others, other types of repeat sequences, including simple repeat, sequences with low complexity. Transposable elements exist in at least two species including mouse were annotated with character A-K.

**Figure 5:**
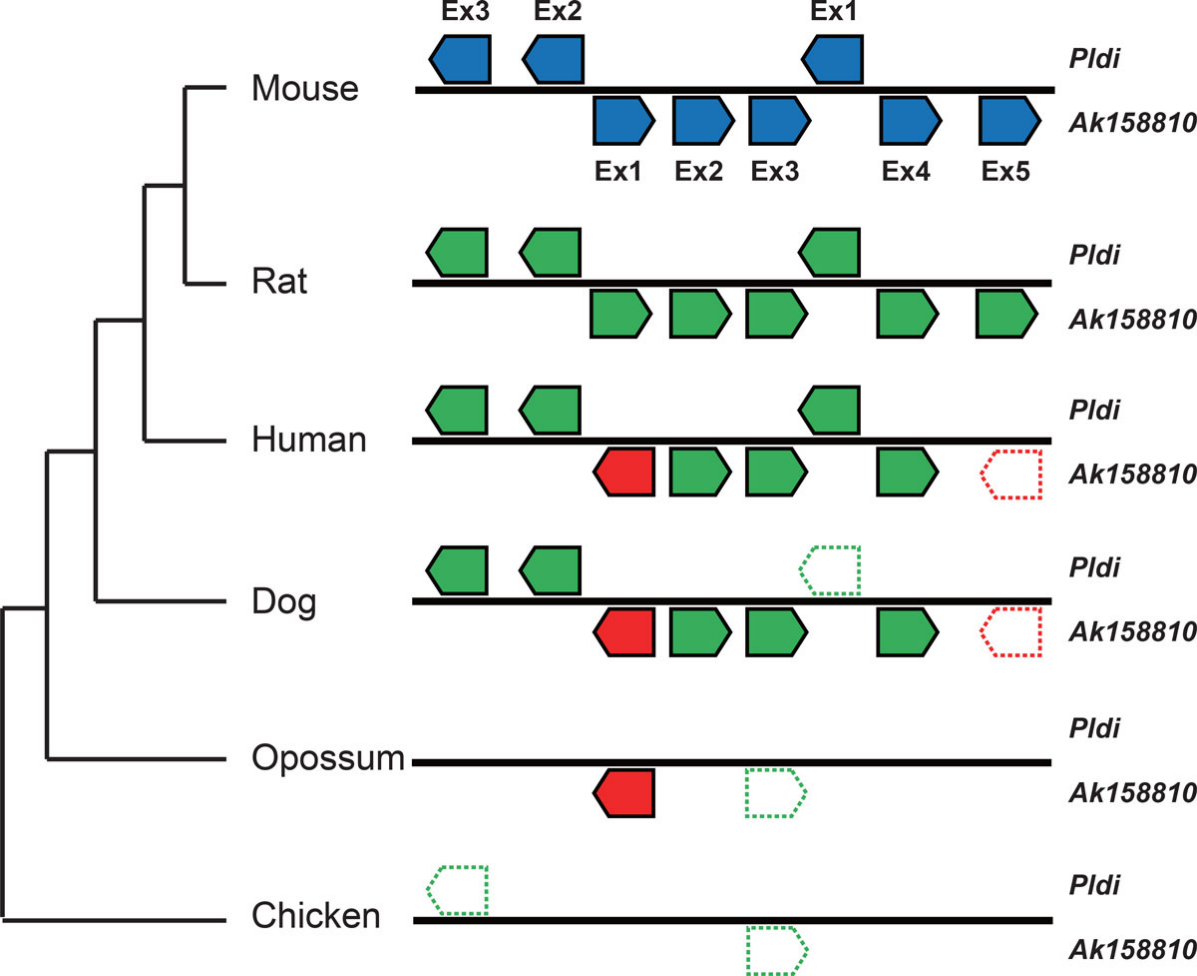
**Formation of *Pldi-Ak158810 *exon sequences**. Green pentagons represent for homologue sequences match with specific mouse exons, red ones represent for inversed exons. Pentagons in bold lines suggest homologues with low similarity or low percent of coverage.

Based on the exons and introns of *Pldi *and *Ak158810*, we further analyzed the origin of them in mouse, rat, human, dog, opossum and chicken (Figure 4 & Figure 5). *Pldi *consists of 3 exons and 2 introns, and *Ak158810 *consists of 5 exons and 4 introns. In non-rodent species, two fragments in intron 1 of *Pldi *were detected. Then, we used MultiZ alignment to compare *Pldi *and its homologues regions in mouse, rat, human, dog, chicken and opossum. We discovered that the majority of the mouse *Pldi*-*Ak158810 *region could be aligned to rat, human, and dog, including exons and introns. In opossum, no fragment was mapped to the three *Pldi *exons. *Ak158810 *exon 1 and part of exon 3 are covered by opossum homologues, matching with the conserved elements detected by our previous analysis (Figure 2). In chicken, few homologue was detected, except partial *Ak158810 *exon 3.

The sequence alignment also revealed that were involved in the evolution of *Pldi *region. We identified two inversions at the loci. First one is the inversion of a ~800 bps fragment, containing the first exon of *Ak158810 *(Figure 5 & Addition File 1). Another inversion is overlapped with *Ak158810 *exon 5. The regions, homologue to the two inversed fragments of non-rodent mammalians, are in opposite direction to those of mouse, which reveals both inversions occurred before the divergence of mouse and rat, and after the divergence of primates and rodents. Interestingly, the first inversed region is highly conserved, which is discussed in following section.

### Transposable events contribute to the formation of *Pldi*-*Ak158810 *loci

Transposable elements (TEs) have been considered as an important composition in the genome [25], we then evaluated the contribution of transposons to the formation of *Pldi*-*Ak158810 *loci. We compared the sequence of mouse *Pldi*-*Ak158810 *loci and homologous sequences in other 5 species (rat, human, dog, opossum and chicken) with the database of mobile elements, using RepeatMasker program [19].To understand whether *Pldi*-*Ak158810 *loci is interrupted by ancient TEs, we manually checked and listed the eleven TEs, which exist in at least two species including mouse (Table 1). No TE was found in opossum and chicken, possibly because of the low homology between this two species and mouse.

**Table 1:** Transposable elements (TEs) that contributed to the formation of ancestral *Pldi-Ak158810 *sequences.

TE symbol	TE name	Repeat Class	Species with the TE
A	MIR	SINE	mouse, rat, human, dog
B	MIR	SINE	mouse, rat, human, dog
C	Chap1_Mam	DNA	mouse, human
D	MER91A	DNA	mouse, rat, human, dog
E	URR1B	DNA	mouse, rat
F	MTEb	LTR	mouse, rat
G	MIR3	SINE	mouse, rat, human
H	MT2B2	LTR	mouse, rat
I	B1F	SINE	mouse, rat
J	PB1D10	SINE	mouse, rat
K	L1MD3	LINE	mouse, human, dog

Only TEs in at least 2 species including mouse are listed. SINE, short interspersed nuclear elements; LINE, long interspersed nuclear elements; DNA, DNA repeat elements; LTR, long terminal repeat elements, including retroposons.

In *Pldi *exon 1 to 3 and *Ak158810 *exon 1 to 4, no ancient TE was detected. However, almost half of all the defined ancient TEs locate in *Ak158810 *exon 5 (Table 2 & Figure 4). Inside the longest *Pldi *intron 1, four ancient TEs were identified, three of which also locate in the overlapped *Ak158810 *intron region (Table 2 & Figure 4). The data shows no evidence that insertions of TEs have been involved in most of the exons at the loci during recent period of time, except for *Ak158810 *exon 5. The formation of the last exon of *Ak158810 *and intron sequences of both *Pldi *and *Ak158810 *are associated with various types of transposable events.

**Table 2:** Gene composition originated from TE

Gene	Element	Origin from TE	
*Pldi*	exon 1		
	exon 2		
	exon 3		
	intron 1	A, B, C, D	
	intron 2		
*Ak158810*	exon 1		
	exon 2		
	exon 3		
	exon 4		
	exon 5	G, H, I, I, K	
	intron 1	B, C	
	intron 2	D	
	intron 3		
	intron 4	E, F	

A, B, C, D, E, F, G, H, I, J, K represent different TE used in Table 1.

### An inverse element is highly conserved and obtains a reduced substitution rate after rearrangement

From genomic sequence in mammals, we noticed the *Pldi*-*Ak158810 *loci was interrupted by chromosome rearrangement in a period of time before its emergence in mouse lineage. Interestingly, one of the rearranged fragments associated with Ak158810 exon 1 is highly conserved among species. From this point of view, we estimated the substitution rate of this highly conserved region among species to test whether inversion contribute to fixation of local region. To better learn the evolution of this loci, we examined the change in substitution rate during the fixation of 4 species, mouse, rat, human and dog. Taking the exon sequences of flanking genes (*pcbd1, slc29a3, sgpl1, unc5b*) as a reference, we constructed a simple model to test the rate change at two time points: the point of chromosome rearrangement and the emergence of *Pldi *and *Ak158810 *in mouse lineage (Figure 5). We extracted sequences of two conserved elements (CE1, conserved element 1 in the inversion; CE2, conserved element 2 near *Ak158810 *exon 3), which could be detected by Blastn in distant organisms.

Compared with surrounding genes (Additional File 7 & Table 3), both CE1 and CE2 obtain the lowest normalized rates during mouse-rat divergence, in line with result from a previous study that purifying selection is acting on *Pldi *region after its emergence [10]. Furthermore, for CE1, the average rate of human-dog divergence is higher than that of human-rat, which implies the substitution rate of this element was slightly reduced after rearrangement. For CE2, not involved in rearranged regions, the tendency is opposite. The data shows the possibility that the specific elements of the *Pldi*-*Ak158810 *loci established their fixation at an early time before the gene birth. Inversion of CE1 may contribute to its acquisition of purifying selection, causing a slightly reduced substitution rate.

**Table 3:** A simplified model to test the change of substitution rate at two time point: occurrence of the inversion and emergence of *Pldi *and *Ak158810 *gene.

Reference	Test sequence	raverHD	raverHR	raverMR
Exons of surrounding genes	Conserved element 1 (inversed region)	2.5971	2.0603	1.406
Exons of surrounding genes	Conserved element 2	1.6694	2.1832	1.5047

Normalized average substitution rates during specific lineage (e.g. "HD" stands for human and dog) are calculated.

## Discussion

### Various factors contribute to the formation of *Pldi-Ak158810 *sequence

A new lncRNA gene could emerge through different scenarios, such as metamorphosis from a protein-coding gene, interrupted by tandem repeat and transposable elements, and *de novo *origin from an intergenic region. Our analysis further confirmed the inter-genic origin of *Pldi*-*Ak158810 *sequence without any clues of long genomic duplication in a recent past. Tracing back in history, both transposable events and chromosome rearrangements were found in the region. In conclusion, the formation of the *Pldi*-*Ak158810 *loci, which became a pair of lncRNA genes in mouse lineage, was affected by multiple factors.

### Fixation of partial *Pldi-Ak158810 *sequence before gene birth

A previous study indicates that the conservation of non-coding RNA is only slightly higher than that of inter-genic region [10]. In *Pldi *region, reduced polymorphism has been detected in specific mouse lineage, which suggests the present of purifying selection. Nevertheless, we found in our data that partial *Pldi*-*Ak158810 *sequence is conserved in all mammalians. It raises the possibility that purifying selection may be acquired in partial *Pldi*-*Ak158810 *region much earlier than the gene birth.

**Figure 6:**
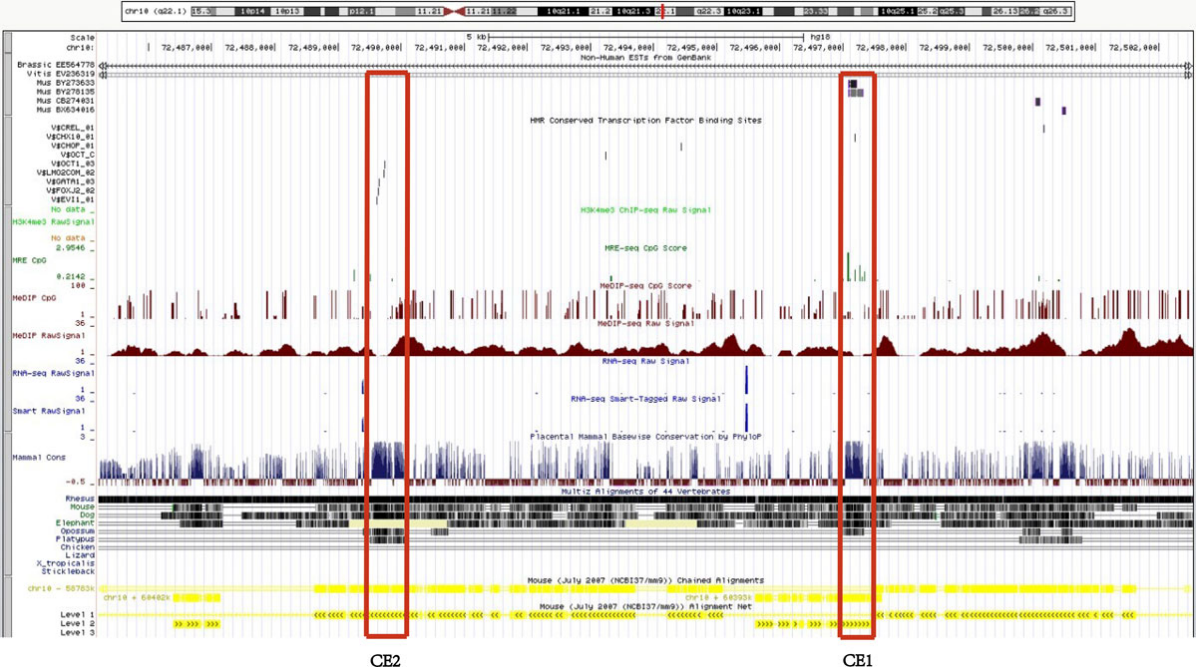
**The demethylation signals and predicted TFBS found in human imply a transcribe tendency in these area**. A strong DNA demethylation signal (green) was found in CE1 region. The HMR conserved TFBS displayed that each black line represents one conserved putative TFBS, which is conserved in human/mouse/rat alignment. The red rectangle demonstrated the consistence of CE1 with potential function signals, like demethylation, TFBS (black) and inversion (yellow arrow direction).

We checked factors that could be responsible to the early fixation. Our calculation of substitution rate change shows that the inversed *Ak158810 *exon 1 was prone to decreasing the evolutional ability after inversion event, relative to surrounding genes (Table 3 & Additional File 7). This trend may represent for an increasing natural selection [25-27]. We also checked DNA modification of the region in human. A series significant signals of demethylation in CpGs are highly correlated with the conserved inversed element, CE1 (Figure 6 & Additional File 3) using Encode browser [28], CE1 is overlapped with the promoter region of *Pldi *'s antisense gene, *Ak158810*, and the promoter sequence in mouse was found with low DNA methylations [21]. Furthermore, from the transcription factor binding site conserved tracks in UCSC, we find this CE1 homologue site is a potential transcription factor binding site of Chx10 conserved in both human and mouse (Figure 6) [24]. This binding site exists both in human and mouse.

According to these observations, we suggested in species other than mouse, partial region of *Pldi*-*Ak158810 *loci could not be simply recognized as "non-functional" before the birth of *Pldi*.

### Birth order of *AK158810 and Pldi*

It has been known that two neighboring genes may form a transcriptional unit [11], which is correlated with expression. As for this case, we assumed the earlier developed lncRNA might influence the birth of the other one by expression level. We attempted to detect the birth order of *AK158810 *and *Pldi*, According to previous studies, the birth order of *Ak158810 *and *Pldi *may not quite clear for the following reasons: first, testis where *Pldi *was born has been considered as an important organ for the emergence of a novel gene [4,29]. According to RNA-seq data (CSHL) and previous study[10], *Pldi *is a testis-specific lncRNA, while *Ak158810 *is likely to have a wide expression range, such as heart, spleen and kidney (Additional File 8). That indicates that *Ak158810 *seems to be a not that young gene as *Pldi *[4,30]; Second, considering northern blot experiment, *Pldi *exists in more species or lineages in mouse testis [10,31], inferring that it is more likely to be older than *Ak158810*. The conflict result, together with the phenomenon, that the expressions of both lncRNAs are limited in mouse, demonstrated that *AK158810 *and *Pldi *were newly transcribed lncRNAs in a similar age after the divergence of mouse and rat.

## Conclusion

In this study, we comprehensively analyzed the sequence origin of a lncRNA antisense gene pair, *Pldi*-*Ak158810*. We found out various factors, including rearrangement, transposable elements, contributed to the formation of the sequence. We also figured out partial sequence of the entire loci is highly conserved in mammalians before the birth and provided evidences and correlated factors for the early fixation of conserved elements.

## Competing interests

The authors declare that they have no competing interests.

## Lists of abbreviation

*Pldi*: Polymorphic derived intron-containing; lncRNA: long non-coding RNA; ESTs: expressed sequence tags; TEs: Transposable elements; CE1: Conserved Element 1; CE2: Conserved Element 2; SINE: Short interspersed nuclear elements; LINE: Long interspersed nuclear elements; DNA: DNA repeat elements; LTR: Long terminal repeat elements, which include retroposons; TFBS: Transcriptional factor binding site; CSHL: Cold Spring Harbor Lab; HMR: Human, mouse, rat.

## Authors' contributions

YL.D., SD.L., X.D., H.S., C.L., Z.L. and BL.Y. carried out all the analysis in this study. X.D. conceived of the study. YL.D., SD.L., X.D., GH.D. and YX.L. wrote the manuscript. All authors read and approved the final manuscript.

## Supplementary Material

Additional file 1 ***Pldi *and its antisense transcript *Ak158810***. It's a screenshot of the region contains *Pldi *and *Ak158810 *from UCSC Browser. These two transcripts share about 8000 bps long. From EST data, there is a potential antisense region overlapped between first exon of *Pldi *and fourth exon of *Ak158810*. Click here for file

Additional file 2 **The subject genomes for Blastn were taken from UCSC**. The genomes of 13 vertebrates were downloaded from UCSC. And the versions of the genome were listed according to the species. Click here for file

Additional file 3 **The Methylation degree of CpGs in *Pldi-Ak158810 *region**. This data was obtained from the forebrain tissue of a lab mouse (GSM809309). The methylation score in y-axis represents the possibility of a CpG site methylated. The x-axis represents the genome position. The arrow showed the direction of the transcript. CpG sites are enriched in the first exon of *Ak158810 *in CE1 region. *Pldi *contains few CpG sites near transcript start region. The blue arrow shows the direction of the transcription. Click here for file

Additional file 4 **The splicing evidence for transcription *Ak158810***. We Compared *Ak158810*, including its introns with mouse EST database in NCBI. Several tags could be mapped to *Ak158810 *exons (in blue cycles). And the first splicing site between exon 1 and exon 2 could be observed. Click here for file

Additional file 5 **Potential ORF of *AK158810*. **ORF finder demonstrated the potential ORF and its position in the transcript of Ak158810. Green frame represents the potential ORF. Only one potential open reading frames longer than 100 amino acids. And two AUG codons with shorter reading frames (about 70 amino acids) precede this long ORF. Frame site, position and length were demonstrated aside. Click here for file

Additional file 6 **Blastn result for *Pldi-Ak158810 *region in 13 species**. Sequence in *Pldi-Ak158810 *region is used as an inquiry to detect the homologue sequence in 12 other vertebrates by Blastn. The command for Blastn is 'blastn -db $db -task blastn -db $db -task megablast -query $query -out $out -outfmt 6'. The length of initial exact match is at least bigger than 28. 'db' is the reference genome of 12 species. 'query' is the sequence of *Pldi *and *Ak158810 *loci in mouse. Click here for file

Additional file 7 **Sequence alignment file of 4 key species**. Alignment files contained the regions we picked from the multiz file of 30 vertebrates to do alignment among dog, human, mouse and rat to calculate the substitution rate in Table 3 based on the model raised in the Figure 1. The alignment file includes inversed element (Conserved element 1), tandem elements (Conserved element 2) and surrounding genes. Click here for file

Additional file 8 **Rna-seq in different tissues of Mouse show this *Pldi *and *Ak158810 *loci is a dynamic transcriptional state in different tissues**. Long RNA-seq data from Encode CSHL provided the expression level of *Pldi-Ak158810 *region in different tissues of mouse from UCSC Browser. wide expression signals of *Pldi *and *Ak158810 *were found in testis. In heart, kidney and spleen, similar transcripts in region of *Ak158810 *were enriched. Other tissues did not show specific expression of these two transcripts. Click here for file

## Declarations

Funding for the publication fee comes from National Natural Science Foundation of China (grant number: 31070752).

This article has been published as part of *BMC Genomics *Volume 14 Supplement 8, 2013: Selected articles from the International Conference on Intelligent Biology and Medicine (ICIBM 2013): Genomics. The full contents of the supplement are available online at http://www.biomedcentral.com/bmcgenomics/supplements/14/S8.
