# Supplementary material for: The de novo sequence origin of two long non-coding genes from an inter-genic region
Source: BMC Genomics. 2013 Dec 9;14(Suppl 8):S6. doi: 10.1186/1471-2164-14-S8-S6 (PMC4042238; doi:10.1186/1471-2164-14-S8-S6)
Supplement: Additional file 1 — Pldi and its antisense transcript Ak158810. It's a screenshot of the region contains Pldi and Ak158810 from UCSC Browser. These two transcripts share about 8000 bps long. From EST data, there is a potential antisense region overlapped between first exon of Pldi and fourth exon of Ak158810. [file 1471-2164-14-S8-S6-S1.PDF]

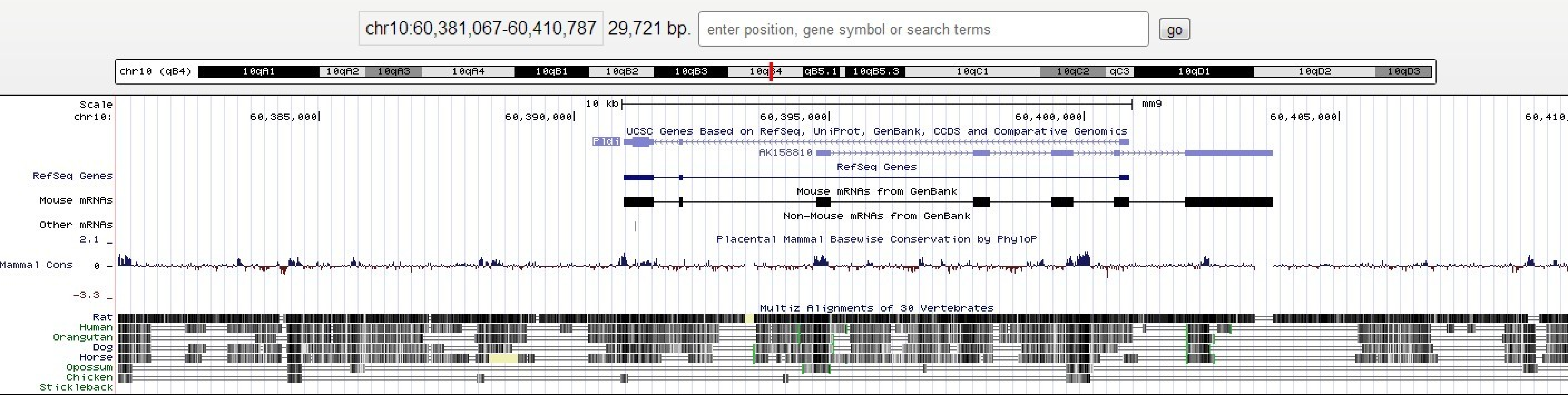
chr10:60,381,067-60,410,787 29,721 bp.
enter position, gene symbol or search terms
go
chr10 (qB4)
10qA1
10qA2
10qA3
10qA4
10qB1
10qB2
10qB3
10qB4
qB5.1
10qB5.3
10qC1
10qC2
qC3
10qD1
10qD2
10qD3
Scale
10 kb
mm9
chr10:
60,385,000
60,390,000
60,395,000
60,400,000
60,405,000
UCSC Genes Based on RefSeq, UniProt, GenBank, CCDS and Comparative Genomics
Pldi
AK158810
RefSeq Genes
RefSeq Genes
Mouse mRNAs
Mouse mRNAs from GenBank
Other mRNAs
Non-Mouse mRNAs from GenBank
2.1
Placental Mammal Basewise Conservation by PhyloP
Mammal Cons
0
-3.3
Multiz Alignments of 30 Vertebrates
Rat
Human
Orangutan
Dog
Horse
Opossum
Chicken
Stickleback
