# Supplementary figures and images for: The de novo sequence origin of two long non-coding genes from an inter-genic region
Source: BMC Genomics. 2013 Dec 9;14(Suppl 8):S6. doi: 10.1186/1471-2164-14-S8-S6 (PMC4042238; doi:10.1186/1471-2164-14-S8-S6)

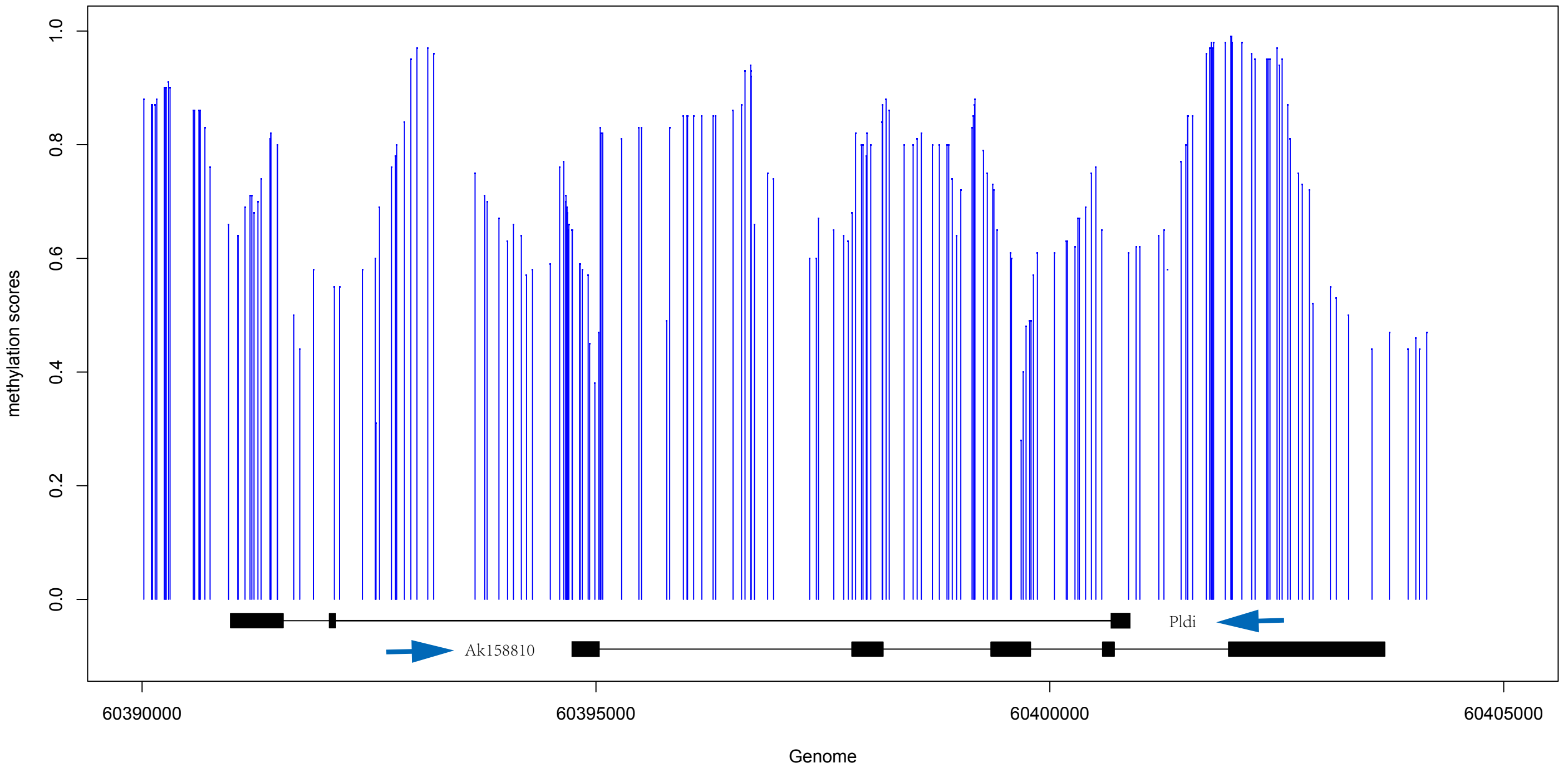

Supplement: Additional file 3 — The Methylation degree of CpGs in Pldi-Ak158810 region. This data was obtained from the forebrain tissue of a lab mouse (GSM809309). The methylation score in y-axis represents the possibility of a CpG site methylated. The x-axis represents the genome position. The arrow showed the direction of the transcript. CpG sites are enriched in the first exon of Ak158810 in CE1 region. Pldi contains few CpG sites near transcript start region. The blue arrow shows the direction of the transcription. [file 1471-2164-14-S8-S6-S3.PDF]

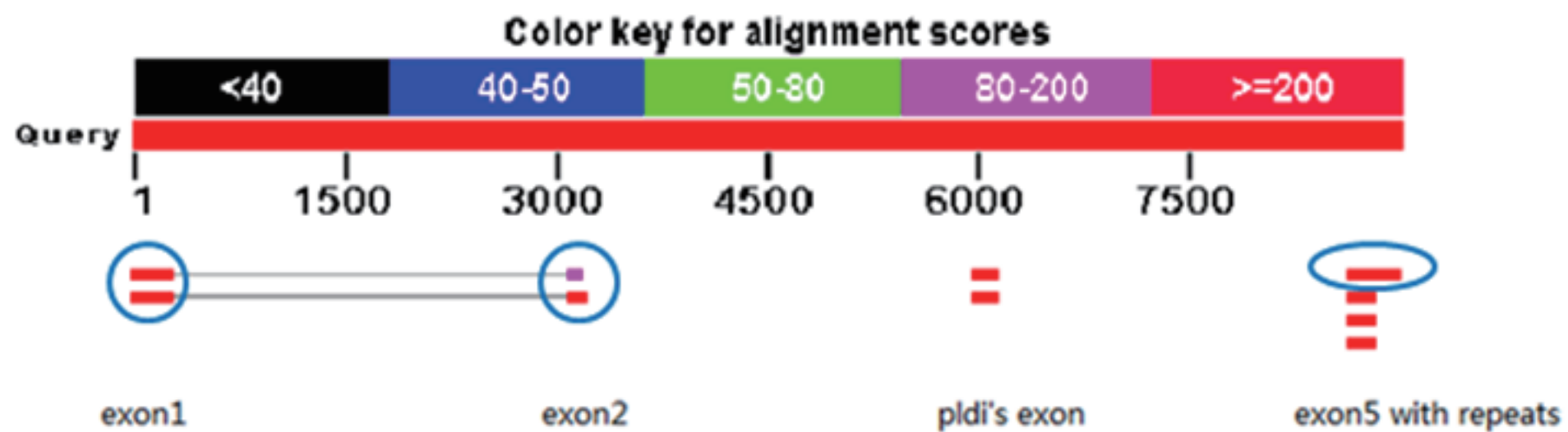

Supplement: Additional file 4 — The splicing evidence for transcription Ak158810. We Compared Ak158810, including its introns with mouse EST database in NCBI. Several tags could be mapped to Ak158810 exons (in blue cycles). And the first splicing site between exon 1 and exon 2 could be observed. [file 1471-2164-14-S8-S6-S4.PDF]

chr10:60,381,067-60,410,787 29,721 bp.

go

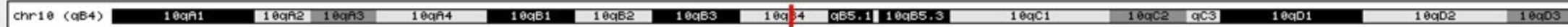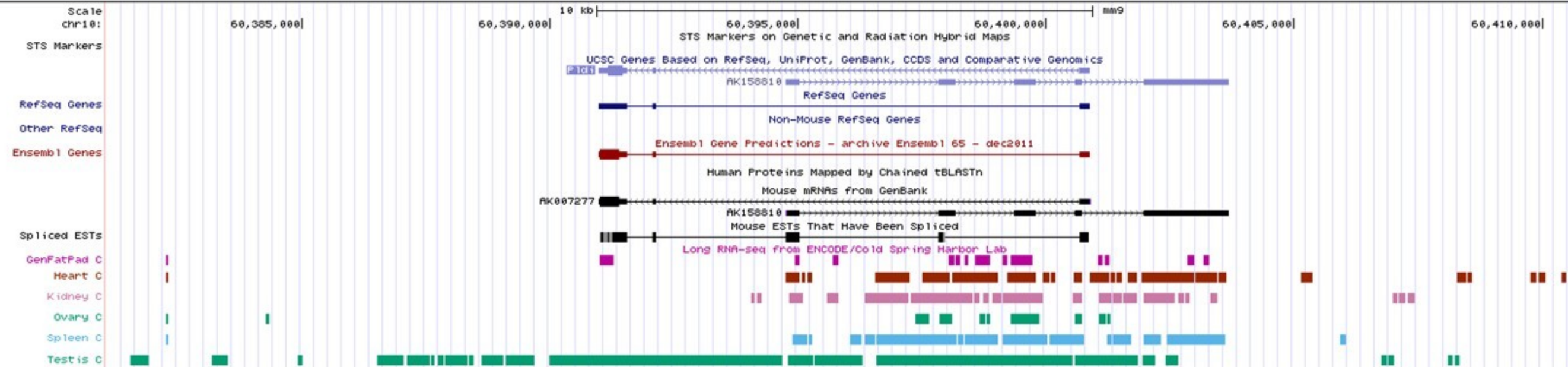

Supplement: Additional file 8 — Rna-seq in different tissues of Mouse show this Pldi and Ak158810 loci is a dynamic transcriptional state in different tissues. Long RNA-seq data from Encode CSHL provided the expression level of Pldi-Ak158810 region in different tissues of mouse from UCSC Browser. wide expression signals of Pldi and Ak158810 were found in testis. In heart, kidney and spleen, similar transcripts in region of Ak158810 were enriched. Other tissues did not show specific expression of these two transcripts. [file 1471-2164-14-S8-S6-S8.PDF]
