# Supplementary material for: The de novo sequence origin of two long non-coding genes from an inter-genic region
Source: BMC Genomics. 2013 Dec 9;14(Suppl 8):S6. doi: 10.1186/1471-2164-14-S8-S6 (PMC4042238; doi:10.1186/1471-2164-14-S8-S6)
Supplement: Additional file 5 — Potential ORF of AK158810. ORF finder demonstrated the potential ORF and its position in the transcript of Ak158810. Green frame represents the potential ORF. Only one potential open reading frames longer than 100 amino acids. And two AUG codons with shorter reading frames (about 70 amino acids) precede this long ORF. Frame site, position and length were demonstrated aside. [file 1471-2164-14-S8-S6-S5.PDF]

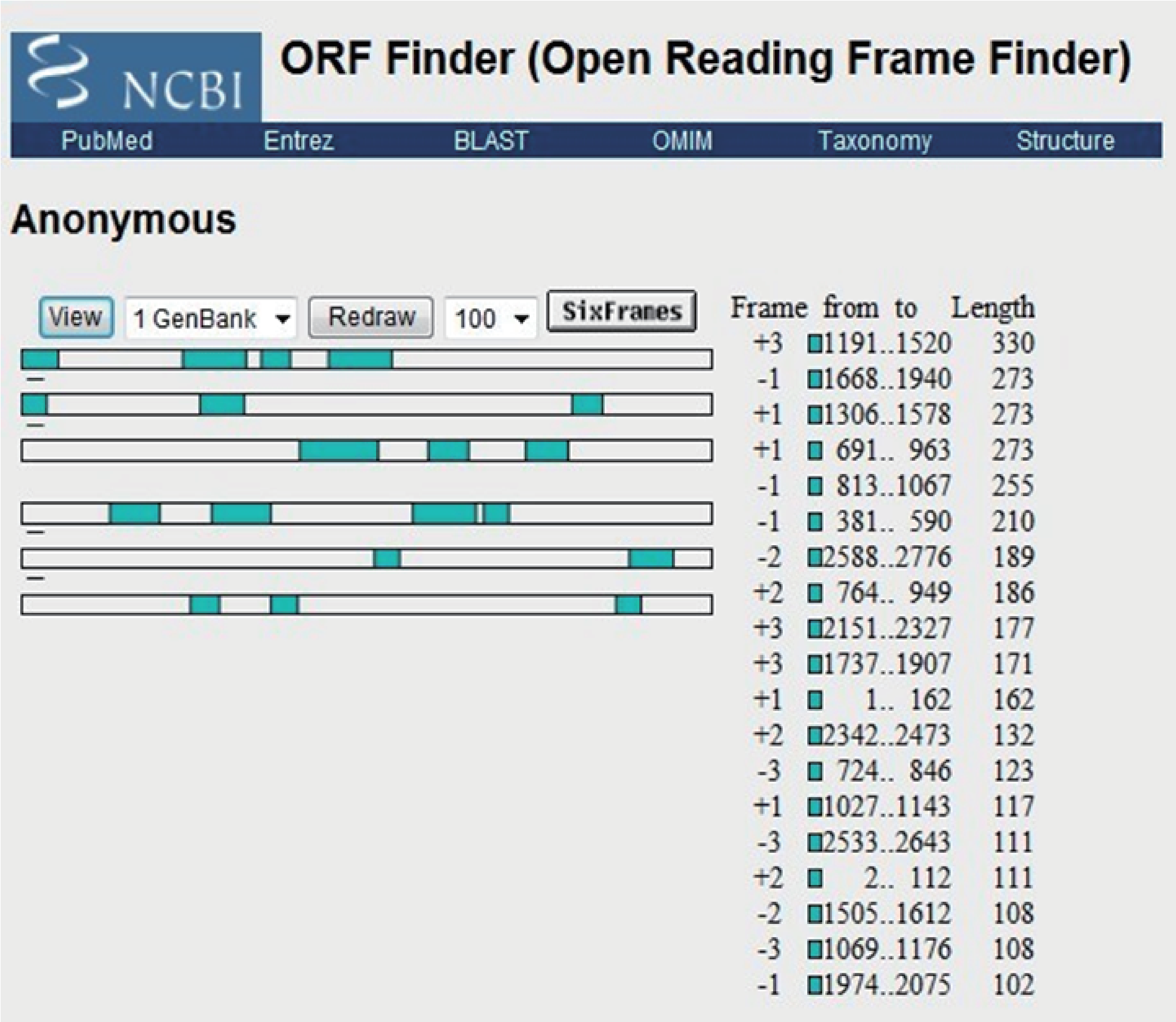
NCBI
ORF Finder (Open Reading Frame Finder)
PubMed
Entrez
BLAST
OMIM
Taxonomy
Structure
Anonymous
View
1 GenBank
Redraw
100
SixFrames
Frame from to Length
+3 1191..1520 330
-1 1668..1940 273
+1 1306..1578 273
+1 691.. 963 273
-1 813..1067 255
-1 381.. 590 210
-2 2588..2776 189
+2 764.. 949 186
+3 2151..2327 177
+3 1737..1907 171
+1 1.. 162 162
+2 2342..2473 132
-3 724.. 846 123
+1 1027..1143 117
-3 2533..2643 111
+2 2.. 112 111
-2 1505..1612 108
-3 1069..1176 108
-1 1974..2075 102
