# Supplementary material for: The de novo sequence origin of two long non-coding genes from an inter-genic region
Source: BMC Genomics. 2013 Dec 9;14(Suppl 8):S6. doi: 10.1186/1471-2164-14-S8-S6 (PMC4042238; doi:10.1186/1471-2164-14-S8-S6)
Supplement: Additional file 7 — Sequence alignment file of 4 key species. Alignment files contained the regions we picked from the multiz file of 30 vertebrates to do alignment among dog, human, mouse and rat to calculate the substitution rate in Table 3 based on the model raised in the Figure 1. The alignment file includes inversed element (Conserved element 1), tandem elements (Conserved element 2) and surrounding genes. [file 1471-2164-14-S8-S6-S7.docx]

inverse element(Conserved element 1)

Mouse Rat human dog

60394420 60395055 28296267 28296833 72496643 72497322 25080438 25081103

tandem element(Conserved element 2)

Mouse Rat human dog

60398787 60400298 28300510 28302009 72491002 72489341 25075535 25073987

Alignment of inversed elements

>DOG_E

-------AAAGGATGACAGAGGTGCATCC-----TGGGGACCCGTTCTCTAACACGGAGC

CTCCCGGGGCTGGGCCAGGAGTTCAGCTCTCATCTACCT-GTCCCCTGTCCCCAAGCCTG

GGAGAAGTCCTTGAAGTCCAGAGTGAGCCCTTGCA-CACGGTGATTTTGTCACACTTCCC

CTGTCCTCCTGGGGCGGG-ATGAGAGGGAGGGGACCGCTGTGAGCAGGAGCACGTCAGCG

TCACTCCACGGTGAGGGCCATTCGGACCTCCTGGAGGAGTCAGAGGCAGCCCAGCTCTGG

GGACGCGC---TGCCCCATCCTGAGTGACAGCAGGC-CAGGGCCCGCAGCCACGGCCACC

GGTGAGCGCCCCGCGGCCCCCGCGAGTGCACACGCTCGCCGCACGTGCGCATGATATTAA

AGTCATATTTTTGGTGGCTGGGAAGGCGCGGTTGTCACTTCCTCTGCCCCAGGCTACCCG

CCG-CCCGGCTCCCCTTCCACTTCCCCGCCC-ACCGCG-AGC-------GCCATCCGTTT

GTATTTATATAGATAAATCGGCCGG-CTAATTAAAGTTCTCTTGCCGGAGAGTGAGGGAA

TTAATAAAAGAACTTGGAACGGAGCCGACGGAAAGCTGGGGCGCTCGGAGCCGGAGCCAG

AAGCCGCGGCAACAAAGGAGGCCACAGGGTCCAGCA------------------------

-----------------------------------------------

>HUMAN_E

CCACGTGTAAGCAAGACAGAGGTGCATCCAACACTTAGGGCCGGTTCTCCAACACTGAGT

CTCCTGAGCCTGGACT-GGAATTGAGCTGTCATTGGGCTCATCCTCCTGCCCCAATCCTG

GTAAAAGTCCTGGAAGTCCAAAGGGAGCCTTTGGAACAGGGTGACTTTGTCATGTGTCCC

CTGCCTCCCTGGGGGAGGTGTGAGAGGGAGGGGAGTTCGATGAGTGGGAATGTGTCAATG

TCGCTCCTAGGTGAGGAG-ATTAAGACCTTCCTGGGAAAGCAGAAACAGTCCAGCTCTGG

AAACATGT---TCCCCATCACACGGTGACAGCCTGCACCCGACCTGCAGACACTGCTCTA

CCAGCGCCATCAGCCAGCCCAGAAAGCGCACACACCCAC-ACACGTGTGGGTGATATTAA

AGTCATATTTTTGGTTGCTGGGGAGGCGCGGTCGTCACTTCCCCTGCCCCAGGCAACCCG

CTG-CC-AGCTCCCCTTTTACTTCCCCACCCCACCGTGGAGT-------GCTTGCCGTTT

GTATTTATATAGATAAATCGGCCGGGCTAATTAAAGTTCTCTTGCTGGAGAGTGAGGGAA

TTAATAAAAGAACTTGGAAAGGAGCCAACTGAAAGCTGGGGCGCTCAGAGCTGCAGCCAG

GACCCACTGCA-CAAAGGAGGCCGGAGGATCCAGCA------------------------

-----------------------------------------------

>MOUSE_E

------------------------------------------------------------

--------------------GTCTA--TGTAAGCAAGACAGAGTGGTTGCCCAGTCTCCT

GCCTGAGTCCTAGAA-TCCAGAGGCAGCTTTTGAA-CATGGTCGCTTTGTCACCTATCCC

TTGCCACCCTAAGGAAGG-----------GGGCTCTGCATCAGGTGGGGACTCACTGATG

TGGCTTCCAAAAGAAGAG-ACTAGGGCTTTCT-GGGAACGCAGAAG-AACTCAGCTCTGA

AGCCATGTATGTCCCCACCACAGAGCGACAGCAC---------------------TTCTC

AGAACACGCCCGCCCGCCC------GCCCGCACACGC---GCACGTGCACACGATATTAA

AGTCATATTTTTGGTTGCTGGGAAGGCGCCGTTGTCACTTCCTCTGCCCTAGGCAACCTG

CTGGTCCAGCTCCTCTTTCACTTCCCCACCCCACTGTGGAGCACCAGCCGCCATCCGTTT

GTATTTATATAGATAAATCGGCCAGGCTAATTAAAGTGCTCTTGCTGCAG--CAAGAGAA

TTAATAAAAGGACTTGGAATGGAGCCGACCTAAAGCTGGGGCGCTGGGAGTCAGAGCT-G

AACCCTCAGCAGCAAAGGAGGCCAGAAGGGCCAGAAGGCACGTGAGCCACCCCACTCCCT

CTTAGGGGGAGGGGCCAGCAGACACGTGAGTCACCCCCTCGCCCTGG

>RAT_E

------------------------------------------------------------

--------------------GTCCA--TGCAAGTCAGACAGAGTGGTCACCCGGTCTCCT

GTCTGAGTCCTGAAA-TCCAGAGGCAGCTTTTGAA-CGTGGTCACTTTGTCACCTGTCCC

TTGTCACCCTAAGGAAGG-----------GGGCTTTCGATCAGTAGGGGACTCACTGATG

TGGCTTTCAAAAGAAGAG-ACCAGGTCTCTCT-GGGAACACAGAAG-AACTCAGCTCTGA

AGCCGTGTGTGACCCCACCACAGAGCGACAGCTC---------------------TTCTC

AGAACATGCCCGTCCGCCC------GCACACACGCACAT-GCACATGCACACGATATTAA

AGTCATATTTTTGGTTGCTGGGAAGGCGCGGTTGTCACTTCCCCTGCCCTAGGCAACCTG

CTGGTCCAGCTCCCCTTTCACTTCCCCGCCCCACTGTGGAGCACCAGCCACCATTCGCTT

GTATTTATATAGATAAATCGGCCGGGCTAATTAAAGTGCTCTTGCTGCAG--TGAGAGAA

TTAATAAAAGAACTTGGAATGGAGCCGACCTAAAGCTGGGGCGCTGGGAGTCAGAGCT-G

AACCCTTGGCAGCAAAGGAGGCCAGAGGAGCCAGCA------------------------

-----------------------------------------------

Alignments of surrounding genes

>mouse

CCGCACTGGACATGGCCGGCAAGGCACACAGGCTGAGCGCCGAGGAGCGAGACCAGCTGC

TGCCAAACCTGAGGGCTGTGGGGTGGAATGAAGTAGAAGGCCGAGATGCTATCTTCAAGC

AGTTCCATTTTAAAGACTTCAACAGGGCTTTTGGCTTCATGACAAGAGTAGCCCTGCAGG

CTGAAAAGCTGGACCACCATCCCGAGTGGTTTAACGTGTACAACAAGGTCCATATCACCT

TGAGCACCCATGAATGTGCCGGTCTTTCGGAACGGGATATAAACCTGGCCAGCTTCATCG

AACAAGTCGCCGTGTCTATGACATAGATC-TACCCTGACTCTT--ATTTGCTTGGGGGAA

-------GGAGTGACTGGAGGAGGAACCTAGGAAGGGAACCAAGGAGGC-T

GTTTATGGTCTCGGT

TTAAAAAAAAATTATTTAAGCTTATCAGGAAGATGCCATTTATTGGACGTAAGATCGAAC

AACAGGTGAGCAAAGCCAAGAAGGATCTTGTCAAGAACATGCCATTCCTAAAGGTGGACA

AGGATTATGTGAAAACTCTGCCTGCTCAGGGTATGGGCACAGCTGAGGTTCTGGAGAGAC

TCAAGGAGTACAGCTCCATGGATGGTTCCTGGCAAGAAGGGAAAGCCTCAGGAGCTGTGT

ACAATGGGGAACCGAAGCTCACGGAGCTGCTGGTGCAGGCTTATGGAGAATTCACGTGGA

GCAATCCACTGCATCCAGATATCTTCCCTGGATTGCGGAAGTTAGAGGCAGAAATCGTTA

GGATGACTTGTTCCCTCTTCAATGGGGGACCAGATTCCTGTGGATGTGTGACTTCTGGGG

GAACGGAAAGCATCCTGATGGCCTGCAAAGCTTACCGGGACTTGGCGTTAGAGAAGGGGA

TCAAAACTCCAGAAATTGTGGCTCCCGAGAGTGCCCATGCTGCATTCGACAAAGCAGCTC

ATTATTTTGGGATGAAGATTGTCCGAGTTGCACTGAAAAAGAACATGGAGGTGGATGTGC

AGGCAATGAAGAGAGCCATCTCCAGGAACACAGCTATGCTGGTCTGTTCTACCCCACAGT

TTCCTCATGGTGTGATGGATCCTGTCCCCGAAGTGGCCAAGTTGGCTGTCAGATATAAAA

TCCCACTCCATGTGGATGCTTGTCTGGGGGGCTTCCTCATTGTCTTCATGGAGAAAGCAG

GGTACCCACTGGAGAAACCATTTGATTTCCGGGTGAAAGGTGTGACCAGCATTTCAGCAG

ATACTCATAAGTATGGCTATGCTCCTAAAGGTTCATCAGTGGTGATGTACTCTAACGAGA

AGTACAGGACGTACCAGTTCTTTGTTGGTGCAGACTGGCAAGGTGGTGTCTACGCATCTC

CAAGCATAGCTGGCTCACGGCCTGGTGGCATCATTGCAGCCTGTTGGGCCGCCTTGATGC

ACTTCGGTGAGAACGGCTATGTTGAAGCTACCAAACAGATCATCAAAACTGCTCGCTTCC

TGAAGTCAGAACTGGAAAACATCAAAAACATCTTCATTTTCGGTGATCCTCAATTGTCAG

TTATTGCTCTGGGATCCAACGATTTTGACATTTACCGACTATCTAATATGATGTCTGCTA

AGGGGTGGAATTTTAACTACCTGCAGTTCCCAAGAAGCATTCATTTCTGCATTACGTTAG

TACATACTCGGAAGCGAGTGGCGATCCAGTTCCTAAAGGATATCCGGGAATCAGTCACAC

AAATCATGAAGAATCCTAAAGCTAAGACCACAGGAATGGGTGCCATCTATGGCATGGCCC

AGGCAACCATTGACAGGAAGCTGGTTGCAGAAATATCCTCCGTCTTCTTGGACTGCCTTT

ATACTACGGACCCCGTGACTCAGGGCAACCAGATGAACGGTTCTCCAAAGCCCCGCTGA

ATGGCCTTTGCCTCTGAGGACAATGTATACCACAGCTCCAAT

GCTGTCTACAGAGCCCCGAGTAACCATCAAGAAGCTGACCAGGAAGCCCTGCTGGGGAAA

CTACTAGACTACCCAGCCCCGGGCCTGCAGAGGCCAGAGGATCGCTTCAATGGTGCCTAC

ATCATCTTCTTCTGCCTGGGAATTGGGGGCCTACTGCCCTGGAACTTCTTTGTCACTGCT

AAGGAGTACTGGGCATATAAACTCCGAAACTGCTCCAGCCCAGCGTCCGGGGAGGACCCT

GAGGACATGGACATCCTGCCTCCTTCCTTGTGTGTTTCTAGAACTACTTTGAGAGCTACC

TGGCAGTTGCCTCCACTGTGCCCTCCCTGCTGTTCCTGGTGGCTAACTTCCTGCTTGTCA

ACAGGGTCCAGGTGCACGTCCGTGTTCTGGCCTCACTGTCCGTGTCCCTGGCCATCTTCG

TGGTTATGATTGTGCTGGTGAAGGTGGATACTTCCTCCTGGACCCGAGGCTTCTTCAGCC

TCACCATCGCGTGCATGGCCATCATTAGCAGCTCCTCCACCATCTTCAATAGCAGCGTTT

ACGGCCTCACAGGCTCCTTCCCCATGAGGAATGCCCAGGCACTGATATCAGGAGGAGCCA

TGGGAGGGACAGTCAGTGCCGTGGCCTTGCTGGTGGACCTGGCAGCATCCAGTGATGTGC

GGGACAGCACGCTAGCCTTCTTCCTCATGGCAGCAGTCTTCCTTGGGCTCTGTATGGGAC

TCTATCTATTGCTGTCCCAACTGGAGTATGCCAGGTACTACATGAGGCCAGTTGCCCCAG

TTCGAGTGTTTTCTGGTGAAGACAACCCATCCCAGGATGCTCCCAGCGCCTCCTCTGTGG

CCCCTGCATCCAGAGTGATGCACACACCGCCCCTTGGACCCATCCTGAAGAAGACGGCTA

GCCTCGGGTTCTGCGCAGTTTCCCTCTACTTTGTCACGGCCTTCATCATCCCCGCCATCT

CCACCAATATCCAGTCCATGCACAAAGGCACCGGCTCTCCATGGACCTCCAAGTTCTTCG

TGCCCCTCACCGTCTTCCTCCTCTTCAACTTTGCTGACCTCTGCGGCCGACAGGTCACAG

CCTGGATCCAGGTGCCAGGTCCTAGGAGCAAGCTGCTCCCCGGACTGGTGGTCTCTCGGT

TCTGCCTTGTGCCTCTCTTCTTGCTCTGTAACTACCAGCCGCGCTCACACTTGACCAAGG

TGCTTTTCCAGTCGGACATCTACCCAGTGCTTTTCACCTGCCTCCTGGGGCTCAGCAACG

GCTACCTCAGCACGCTGGTGCTCATCTATGGGCCCAAGATTGTGCCCCGGGAGCTGGCTG

AGGCCACCAGTGTTGTGATGTTGTTCTATATGTCTGTGGGCTTGATGCTGGGCTCAGCCT

GCGCGGCCCTGCTTGAACACTTTATC

ATGA

GGGCCCGGAGCGGGGTGCGGAGCGCGCTGCTGCTG---GCGCTGCTGCTTTGCTGGGATC

CGACACCGAGCCTAGCAGGCGTTGACTCTGCTGGCCAGGTGCTCCCAGACTCCTACCCAT

CAGCCCCTGCGGAGCAGCTGCCGTACTTCCTATTGGAGCCACAGGACGCCTACATCGTAA

AGAACAAGCCAGTGGAACTGCACTGCAGAGCCTTCCCTGCCACGCAGATCTACTTCAAGT

GTAATGGCGAGTGGGTCAGCCAGAATGACCACGTCACACAGGAGAGCCTGGATGAGGCCA

CAGGCTTGCGGGTGCGAGAGGTGCAGATCGAGGTGTCACGGCAGCAAGTGGAGGAACTCT

TCGGGCTCGAGGACTACTGGTGCCAGTGCGTGGCCTGGAGCTCTTCGGGAACTACCAAGA

GTCGCCGAGCCTACATCCGCATTGCCTACTTGCGCAAGAACTTTGACCAGGAGCCTCTGG

CCAAGGAGGTACCCTTGGATCATGAGGTCCTTCTGCAGTGCCGCCCACCGGAGGGAGTGC

CTGTGGCTGAGGTGGAATGGCTCAAGAATGAAGATGTCATTGACCCCGCTCAGGACACTA

ACTTCCTGCTCACCATTGACCACAACCTCATCATCCGCCAGGCGCGCCTCTCAGACACGG

CCAACTACACCTGTGTGGCCAAGAATATCGTGGCCAAGCGCCGGAGCACCACGGCCACAG

TCATCGTCTATGTGAATGGAGGCTGGTCCAGCTGGGCAGAGTGGTCACCCTGTTCCAATC

GCTGTGGCCGAGGCTGGCAGAAGCGTACTCGGACCTGCACCAATCCAGCCCCACTCAATG

GAGGCGCCTTCTGTGAGGGACAGGCCTTCCAGAAGACAGCTTGCACCACCGTGTGCCCAG

TGGATGGAGCGTGGACCGAGTGGAGCAAGTGGTCTGCCTGCAGCACAGAGTGTGCGCACT

GGCGCAGCCGCGAGTGCATGGCACCGCCACCCCAGAACGGAGGCCGTGACTGCAGCGGGA

CGCTACTTGACTCCAAGAACTGCACTGATGGGCTGTGCGTGCTGAATCAGAGAACTCTAA

ACGACCCTAAAAGCCACCCCCTGGAGACATCGGGAGATGTGGCACTGTACGCAGGCCTTG

TGGTGGCCGTCTTTGTGGTGGTAGCGGTTCTCATGGCCGTGGGAGTGATCGTATACCGGA

GAAACTGCCGGGACTTCGACACGGACATCACCGACTCCTCTGCGGCCCTCACTGGTGGCT

TCCACCCTGTCAACTTCAAGACTGCAAGGCCCAACAACCCGCAGCTCCTGCACCCGTCCG

CCCCTCCAGACCTAACGGCCAGTGCTGGCATCTACCGCGGGCCTGTGTATGCCCTGCAGG

ACTCCGCCGACAAGATCCCCATGACTAATTCGCCCCTGCTGGATCCCCTGCCCAGCCTCA

AGATCAAGGTCTATAACTCCAGCACCATCGGTTCTGGGTCTGGCCTGGCTGATGGAGCCG

ACCTGCTGGGTGTCCTCCCGCCGGGCACGTACCCAGGCGATTTCTCCCGGGACACCCATT

TCCTGCACCTGCGCAGTGCCAGCCTTGGTTCCCAGCACCTCCTGGGCCTACCTCGGGACC

CCAGCAGCAGTGTCAGCGGCACCTTTGGTTGCCTGGGAGGAAGGCTGAGCCTCCCCGGCA

CAGGGGTCAGCCTGTTGGTACCAAATGGAGCCATTCCCCAGGGCAAGTTCTATGACCTGT

ATCTACATATCAACAAGGCCGAAAGCACCCTCCCACTTTCAGAAGGTTCCCAGACAGTAT

TGAGCCCCTCGGTGACCTGTGGGCCCACAGGCCTACTCCTGTGCCGCCCTGTCGTCCTCA

CCGTGCCCCACTGTGCTGAAGTCATCGCTGGAGACTGGATCTTTCAGCTCAAGACCCAGG

CCCATCAGGGCCACTGGGAGGAGGTGGTGACCTTGGATGAGGAGACCCTCAACACACCCT

GCTACTGCCAGCTGGAGGCTAAGTCCTGCCACATCCTGCTGGACCAGCTGGGTACCTACG

TATTCATGGGCGAGTCCTACTCTCGCTCTGCAGTCAAGCGGCTCCAGCTGGCCATCTTCG

CCCCAGCCCTCTGCACCTCCCTGGAGTATAGCCTCAGGGTCTACTGTCTGGAGGACACAC

CTGTAGCACTGAAGGAGGTCCTGGAGCTGGAGAGGACTCTGGGTGGCTACTTGGTGGAGG

AGCCCAAGCCTTTGCTCTTTAAGGACAGTTACCACAACCTACGCCTCTCCCTCCATGACA

TCCCCCATGCCCACTGGAGGAGCAAACTACTGGCCAAGTACCAGGAGATTCCCTTCTACC

ACGTCTGGAATGGCAGCCAGAGAGCCCTGCACTGCACTTTCACCCTGGAGAGGCATAGCC

TGGCCTCCACGGAGTTCACCTGTAAGGTCTGCGTGCGGCAGGTCGAAGGGGAAGGCCAGA

TTTTCCAGCTGCACACAACGTTGGCCGAGACGCCTGCTGGCTCCCTGGATGCTCTCTGCT

CTGCCCCGGGCAATGCCATCACCACCCAGCTGGGACCCTATGCCTTCAAGATACCCCTGT

CCATCCGCCAAAAGATCTGCAGCAGCCTGGACGCCCCCAACTCCCGGGGCAACGACTGGA

GGCTGTTGGCGCAGAAGCTGTCCATGGACCGGTACCTAAACTACTTCGCCACCAAAGCTA

GTCCCACAGGTGTCATCTTAGACCTCTGGGAAGCTCGGCAACAGGATGACGGGGACCTCA

ACAGCCTGGCCAGTGCCTTGGAGGAGATGGGCAAGAGTGAGATGCTGGTAGCCATGGCCA

CAGATGGCGATTGCTGA

>rat

CCGCACTGGACATGGCTGGCAAGGCACACAGGCTGAGTGCTGAGGAACGGGACCAGCTGC

TGCCAAACCTGCGGGCTGTGGGGTGGAATGAACTGGAAGGCCGAGATGCCATCTTCAAAC

AGTTCCATTTTAAAGACTTCAACAGGGCTTTTGGCTTCATGACAAGAGTCGCCCTGCAGG

CTGAAAAGCTGGACCACCATCCCGAGTGGTTTAACGTGTACAACAAGGTCCATATCACCT

TGAGCACCCACGAATGTGCCGGTCTTTCTGAACGGGATATAAACCTGGCCAGCTTCATCG

AACAAGTTGCCGTGTCTATGACATAGATC-TACCCTGCCTCCT--ATTTCCTTAGGGGAA

AGGAGAAGGAGTGACTGGAGGAGGAACCCAGGGAGGGAACCAAGGAGGC-T

GTTTATGGTCTCGGT

TTAAAAATAAATTATTTAGGCTTATAAGGAAGATGCCATTTATTGGACGTAAGATACAAC

AGCAGCTTACCAAAGCCAAGAAGGATCTTGTCAAGAACATGCCATTCCTGAAGTTGGACA

AAGATTATGTGAAAACCCTGCCTGCTCAGGGTCTGAGCACAGCTGAGGTTCTGGAGAGAC

TCAAGGAGTACAGCTCCATGGATGTCTTCTGGCAAGAAGGGAAAGCCTCAGGAGCTGTGT

ACAGTGGGGAGCCGAAGCTCACCGAGCTGCTGGTGCAGGCTTACGGAGAATTCACGTGGA

GCAATCCACTGCACCCAGATATCTTCCCCGGACTACGGAAGTTAGAGGCAGAAATCGTGA

GGATGACTTGCTCCCTCTTCAATGGGGGGCCAGACTCCTGTGGATGTGTGACTTCTGGGG

GAACAGAAAGCATCCTAATGGCCTGCAAAGCATATCGGGACTTGGCCTTAGAGAAGGGGA

TCAAAACTCCAGAAATTGTGGCTCCTGAGAGTGCCCACGCCGCATTCGACAAAGCAGCTC

ATTATTTCGGAATGAAGATTGTACGCGTTGCGCAGAAAAAGAACATGGAGGTGGATGTGC

GGGCAATGAAAAGAGCCATCTCCAGGAACACAGCCATGCTGGTCTGTTCTGCCCCACAGT

TTCCTCATGGTGTGATAGATCCTATCCCCGAAGTGGCCAAGCTGGCTGTCAAATATAAAA

TCCCATTCCATGTGGATGCTTGTCTTGGGGGCTTCCTCATTGTCTTCATGGAGAAAGCAG

GGTACCCACTGGAGAAACCATTTGATTTCCGGGTGAAAGGTGTGACCAGCATTTCAGCAG

ATACTCATAAGTACGGCTATGCTCCCAAAGGTTCATCAGTGGTGATGTACTCTAACGAGA

AGTACAGGAAGTACCAGTTCTTTGTTGATGCAGATTGGCAAGGTGGCATCTACGCATCTC

CAAGCATAGCTGGCTCACGGCCTGGTGGCATCATTGCAGCCTGTTGGGCCGCCTTGATGC

ACTTTGGTGAGAACGGCTATGTTGAAGCTACCAAACAGATCATCAAAACTGCTCGCTTCC

TCAAGTCAGAACTGGAAAACATCAAAAACATCTTCATTTTGGGGGATCCTCAATTGTCAG

TTATTGCTCTGGGCTCCAACGATTTTGACATCTACCGACTATCTAATATGATGTCTGCTA

AGGGGTGGAATTTTAACTACTTGCAGTTCCCAAGAAGCATTCATTTCTGCATTACGTTAG

TACATACTCGGAAGCGAGTGGCAATCCAGTTCCTAAAGGATATCCGGGAATCGGTCACAC

AAATCATGAAGAATCCTAAAGCTAAGACCACAGGAATGGGTGCCATCTATGGCATGGCCC

AGGCAACCATTGACAGGAAGATGGTTGCAGAAATATCCTCCGTCTTCTTGGACAGCCTTT

ATAGTACAGACCCTGTGACTCAGGGTAACCAGATGAACGGTTCTCCAAAGCCCCGCTGA

ATGGCCTTTGCCTCTGAGGACATTGCATACCACAGCTCAAAT

GCTGTCTACAGAGTCCCAAGCAACCGTCATGAAGCTGACCAGGAAGCCCTACTGGGAAAA

CCACTAGACTACCCAGCCCCAGGCCTGCAGAGGCCAGAGGACCGCTTCAATGGTGCCTAT

ATCATCTTCTTCTGCCTGGGAATTGGCGGCCTACTACCCTGGAACTTTTTTGTCACTGCC

AAAGAGTACTGGGCATTTAAACTCCGAAACTGCTCCAGCCCAGCCTCCGGGAAGGACCCA

GAGGATGCAGACATCCTG-----------------------AACTACTTTGAGAGCTACC

TGGCGGTTGCTTCCACTGTGCCTTCCCTGCTGTTTCTGGTGGCTAACTTCCTGCTTGTCA

ACAGGATCCGGGTGCATGTCCGAGTTCTGGCCTCACTGTCCGTCTCCCTGGCCATCTTTG

TGGTTATGGCCGTGCTGGTGAGGGTGGACACTTCTTCCTGGACCCGGGGCTTCTTCAGCA

TCGCCATGGCGTGCATGGCCATCATCAGCAGCTCCTCCACCATCTTCAATAGCAGCGTGT

ATGGCCTGACGGGCTCGTTCCCCATGAGGAATGCCCAGGCACTGATATCAGGAGGAGCCA

TGGGAGGGACAGTCAGTGCTGTGGCCTCCCTGGTGGACCTGGCAGCATCCAGTGACGTGC

GAGACAGTGCGCTGGCCTTCTTCCTCACAGCAGCAGTCTTCCTTGGGCTCTGTGTGGGGC

TCTACCTACTGCTGCCCCAACTGGAGTATGCCAGGTACTACATGAGGCCGGTTGTCCCAA

TCCACGTGTTTTCTAGTGAAGACAGCCCACCCCGGGATGCTCCCAGCACCTCCTCCGTGG

CCCCTGCATCCAGAGCAGTGCACACGCCACCCCTCGGACCCATCCTGAAGAAGACAGCTG

GCCTGGGGTTCTGCGCCGTTTTCCTCTACTTCATCACCGCCCTTATCTTCCCCGCTATCT

CCACCAACATCCAGCCCATGCACAAGGGCACCGGCTCTCCATGGACCTCCAAGTTCTATG

TGCCCCTCACCGTCTTCCTCCTTTTCAACTTTGCTGACCTCTGCGGCCGACAGGTCACAG

CCTGGATCCAGGTGCCAGGTCCTAGGAGCAAGCTGCTCCCCATACTGGCAGTCTCTCGCG

TCTGCCTCGTGCCTCTCTTCCTGCTCTGTAACTACCAGCCACGCTCACACCTGACTCTGG

TGCTTTTCCAGTCTGACATCTACCCTATACTCTTCACCTGCCTCTTGGGGCTCAGTAATG

GCTACCTCAGCACGCTGGTGCTCATGTATGGGCCCAAGATTGTGCCCCGGGAGCTGGCTG

AGGCCACCAGTGTGGTGATGCTGTTCTACATGTCACTGGGTTTGATGCTGGGCTC-----

--------------------------

ATGA

GGGCCCGGAGCGGGGCGCGGGGCGCGCTGCTGCTGCTGGCGCTGCTGCTCTGCTGGGATC

CGACACCGAGCTTAGCAGGCATTGACTCTGGTGGCCAGGCACTCCCAGACTCCTTCCCAT

CAGCACCCGCGGAGCAGCTGCCTCACTTCCTGCTGGAACCAGAGGATGCCTACATCGTAA

AGAACAAGCCAGTGGAATTGCACTGCCGAGCCTTCCCTGCCACACAGATCTACTTCAAGT

GTAATGGCGAGTGGGTTAGCCAGAAAGGCCACGTCACGCAGGAGAGCCTGGATGAGGCCA

CAGGCTTGCGAATACGAGAGGTGCAGATAGAGGTGTCGCGGCAGCAGGTGGAGGAACTCT

TTGGGCTCGAGGACTACTGGTGTCAGTGCGTGGCCTGGAGCTCTTCGGGAACCACCAAGA

GTCGCCGAGCCTACATCCGCATTGCCTACTTGCGCAAGAACTTTGACCAGGAGCCTCTGG

CGAAGGAGGTACCCTTGGATCATGAGGTCCTTCTGCAGTGCCGCCCACCAGAGGGAGTGC

CTGTGGCTGAGGTGGAATGGCTCAAGAATGAAGATGTCATCGATCCCGCTCAGGACACTA

ACTTCCTGCTCACCATTGACCACAACCTCATCATCCGCCAGGCGCGCCTCTCAGACACAG

CCAACTACACCTGTGTGGCCAAGAATATTGTGGCCAAGCGCCGGAGCACGACGGCCACAG

TCATCGTCTATGTGAACGGAGGTTGGTCCAGCTGGGCAGAATGGTCACCCTGCTCTAACC

GCTGCGGCCGAGGTTGGCAGAAACGTACTAGGACCTGCACCAACCCAGCCCCACTCAATG

GAGGTGCCTTCTGCGAGGGACAGGCTTTCCAGAAGACGGCTTGCACCACCGTGTGCCCAG

TGGATGGAGCGTGGACTGAGTGGAGCAAGTGGTCCGCCTGCAGCACAGAGTGTGCGCACT

GGCGCAGCCGCGAGTGCATGGCACCGCCGCCCCAGAACGGAGGCCGCGACTGCAGCGGGA

CGCTACTTGACTCCAAGAACTGCACCGATGGGCTGTGCGTGCTGAATCAGAGAACTCTAA

ACGACCCTAAAAGCCGCCCCCTGGAGCCGTCGGGAGACGTGGCGCTGTATGCGGGCCTCG

TGGTGGCCGTCTTTGTGGTTCTGGCAGTTCTCATGGCTGTAGGAGTGATCGTGTACCGGA

GAAACTGCCGGGACTTCGACACGGACATCACTGACTCCTCTGCTGCCCTCACTGGTGGTT

TCCACCCCGTCAACTTCAAGACTGCAAGGCCCAGCAACCCACAGCTCCTGCACCCATCCG

CCCCTCCGGACCTAACGGCCAGTGCTGGCATCTACCGCGGACCTGTGTATGCCCTGCAGG

ACTCCGCCGACAAGATCCCTATGACTAATTCACCCCTGCTGGATCCCTTGCCCAGCCTCA

AGATCAAGGTCTATGACTCCAGCACCATCGGCTCTGGGGCTGGCCTGGCTGATGGAGCCG

ACCTGCTGGGTGTCTTACCACCCGGTACATACCCAGGCGATTTCTCCCGGGACACCCACT

TCCTGCACCTGCGCAGCGCCAGCCTCGGTTCCCAGCACCTCCTGGGCCTCCCTCGAGACC

CCAGCAGCAGTGTCAGTGGCACCTTTGGTTGCCTGGGTGGGAGGCTGACCATTCCCGGCA

CAGGGGTCAGCCTGTTGGTACCAAATGGAGCCATTCCCCAGGGCAAGTTCTATGACTTGT

ATCTACGTATCAACAAGACTGAAAGCACCCTCCCACTTTCAGAAGGTTCCCAGACAGTAT

TGAGCCCCTCGGTGACCTGCGGGCCCACGGGCCTCCTCCTGTGCCGCCCTGTTGTCCTCA

CTGTGCCCCACTGTGCTGAAGTCATTGCCGGAGACTGGATCTTCCAGCTCAAGACCCAGG

CCCATCAGGGCCACTGGGAGGAGGTGGTGACTTTGGATGAGGAGACTCTGAACACCCCCT

GCTACTGCCAGCTAGAGGCTAAATCCTGCCACATCCTGTTGGACCAGCTGGGTACCTACG

TGTTCACGGGCGAGTCCTACTCCCGCTCCGCAGTCAAGCGGCTCCAGCTAGCCATCTTCG

CCCCAGCCCTCTGCACCTCCCTGGAGTATAGTCTCAGGGTCTACTGTCTGGAGGACACTC

CTGCAGCACTGAAGGAGGTCCTAGAACTGGAGAGGACTCTGGGTGGCTACTTGGTGGAGG

AGCCCAAGCCTTTGCTCTTTAAGGACAGTTACCACAACCTACGCCTCTCCCTCCATGACA

TCCCCCATGCCCACTGGAGGAGCAAACTACTGGCCAAGTACCAGGAGATTCCCTTCTACC

ATGTGTGGAACGGCAGCCAGAAAGCCCTGCACTGCACTTTCACCCTGGAGAGACATAGCC

TAGCCTCCACTGAGTTCACCTGTAAGGTCTGCGTGCGGCAGGTAGAAGGGGAAGGCCAGA

TTTTCCAGCTGCACACCACGCTGGCTGAGACGCCTGCTGGCTCCCTGGATGCACTCTGCT

CTGCCCCTGGCAATGCTGCCACCACACAGCTGGGACCCTATGCCTTCAAGATACCACTGT

CCATCCGCCAGAAGATCTGCAACAGCCTGGACGCCCCCAACTCACGGGGCAATGACTGGC

GGCTGTTGGCACAGAAGCTCTCCATGGACCGGTACCTGAACTACTTCGCCACCAAAGCTA

GTCCCACAGGCGTGATCTTAGACCTCTGGGAAGCTCGGCAGCAGGATGATGGGGACCTCA

ACAGCCTGGCCAGTGCCTTGGAGGAGATGGGCAAGAGTGAGATGCTGGTAGCCATGACCA

CTGATGGCGATTGCTGA

>human

CCGCCCGCGCCATGGCTGGCAAAGCACACAGGCTGAGCGCTGAGGAGAGGGACCAGCTGC

TGCCAAACCTGAGGGCTGTGGGGTGGAATGAGCTGGAAGGCCGTGATGCCATCTTCAAGC

AGTTTCATTTCAAAGACTTCAACAGGGCCTTTGGGTTCATGACAAGAGTGGCCCTGCAGG

CTGAGAAACTGGACCACCATCCTGAATGGTTTAACGTGTACAACAAGGTCCACATCACGC

TGAGCACCCATGAGTGTGCCGGCCTTTCAGAACGGGACATAAACCTGGCCAGCTTCATCG

AACAAGTAGCAGTGTCCATGACATAGACCCTGCCCTTCCTCTTTGAATTCTTCCGGGGGA

A------GGGGTGACTGAACTGGGAGTCCAGGGAGGGAGCTGAGGAGCCCT

GTTTATGGTCAAGGT

TTAAAAAGAAATGTTTTAAGCTCACCAGGAAGATGCCCATTATTGGTCGTAAGATTCAAG

ACAAGTTGAACAAGACCAAGGATGATATTAGCAAGAACATGTCATTCCTGAAAGTGGACA

AAGAGTATGTGAAAGCTTTACCCTCCCAGGGTCTGAGCTCATCTGCTGTTTTGGAGAAAC

TTAAGGAGTACAGCTCTATGGACGCCTTCTGGCAAGAGGGGAGAGCCTCTGGAACAGTGT

ACAGTGGGGAGGAGAAGCTCACTGAGCTCCTTGTGAAGGCTTATGGAGATTTTGCATGGA

GTAACCCCCTGCATCCAGATATCTTCCCAGGACTACGCAAGATAGAGGCAGAAATTGTGA

GGATAGCTTGTTCCCTGTTCAATGGGGGACCAGATTCGTGTGGATGTGTGACTTCTGGGG

GAACAGAAAGCATACTGATGGCCTGCAAAGCATATCGGGATCTGGCCTTTGAGAAGGGGA

TCAAAACTCCAGAAATTGTGGCTCCCCAAAGTGCCCATGCTGCATTTAACAAAGCAGCCA

GTTACTTTGGGATGAAGATTGTGCGGGTCCCATTGACGAAGATGATGGAGGTGGATGTGC

GGGCAATGAGAAGAGCTATCTCCAGGAACACTGCCATGCTCGTCTGTTCTACCCCACAGT

TTCCTCATGGTGTAATAGATCCTGTCCCTGAAGTGGCCAAGCTGGCTGTCAAATACAAAA

TACCCCTTCATGTCGACGCTTGTCTGGGAGGCTTCCTCATCGTCTTTATGGAGAAAGCAG

GATACCCACTGGAGCACCCATTTGATTTCCGGGTGAAAGGTGTAACCAGCATTTCAGCTG

ACACCCATAAGTATGGCTATGCCCCAAAAGGCTCATCATTGGTGTTGTATAGTGACAAGA

AGTACAGGAACTATCAGTTCTTCGTCGATACAGATTGGCAGGGTGGCATCTATGCTTCCC

CAACCATCGCAGGCTCACGGCCTGGTGGCATTAGCGCAGCCTGTTGGGCTGCCTTGATGC

ACTTCGGTGAGAACGGCTATGTTGAAGCTACCAAACAGATCATCAAAACTGCTCGCTTCC

TCAAGTCAGAACTGGAAAATATCAAAGGCATCTTTGTTTTTGGGAATCCCCAATTGTCAG

TCATTGCTCTGGGATCCCGTGATTTTGACATCTACCGACTATCAAACCTGATGACTGCTA

AGGGGTGGAACTTGAACCAGTTGCAGTTCCCACCCAGTATTCATTTCTGCATCACATTAC

TACACGCCCGGAAACGAGTAGCTATACAATTCCTAAAGGACATTCGAGAATCTGTCACTC

AAATCATGAAGAATCCTAAAGCGAAGACCACAGGAATGGGTGCCATCTATGGCATGGCCC

AGACAACTGTTGACAGGAATATGGTTGCAGAATTGTCCTCAGTCTTCTTGGACAGCTTGT

ACAGCACCGACACTGTCACCCAGGGCAGCCAGATGAATGGTTCTCCAAAACCCCACTGA

ATGGCCGTTGTCTCAGAGGACGACTTTCAGCACAGTTCAAAC

TCCACCTACAGAACCACAAGCAGCAGTCTCCGAGCTGACCAGGAGGCACTGCTTGAGAAG

CTGCTGGACCGCCCGCCCCCTGGCCTGCAGAGGCCCGAGGACCGCTTCTGTGGCACATAC

ATCATCTTCTTCAGCCTGGGCATTGGCAGTCTACTGCCATGGAACTTCTTTATCACTGCC

AAGGAGTACTGGATGTTCAAACTCCGCAACTCCTCCAGCCCAGCCACCGGGGAGGACCCT

GAGGGCTCAGACATCCTG-----------------------AACTACTTTGAGAGCTACC

TTGCCGTTGCCTCCACCGTGCCCTCCATGCTGTGCCTGGTGGCCAACTTCCTGCTTGTCA

ACAGGGTTGCAGTCCACATCCGTGTCCTGGCCTCACTGACGGTCATCCTGGCCATCTTCA

TGGTGATAACTGCACTGGTGAAGGTGGACACTTCCTCCTGGACCCGTGGCTTTTTTGCGG

TCACCATTGTCTGCATGGTGATCCTCAGCGGTGCCTCCACTGTCTTCAGCAGCAGCATCT

ACGGCATGACCGGCTCCTTTCCTATGAGGAACTCCCAGGCACTGATATCAGGAGGAGCCA

TGGGCGGGACGGTCAGCGCCGTGGCCTCATTGGTGGACTTGGCTGCATCCAGTGATGTGA

GGAACAGCGCCCTGGCCTTCTTCCTGACGGCCACTGTCTTCCTCGTGCTCTGCATGGGAC

TCTACCTGCTGCTGTCCAGGCTGGAGTATGCCAGGTACTACATGAGGCCTGTTCTTGCGG

CCCATGTGTTTTCTGGTGAAGAGGAGCTTCCCCAGGACTCCCTCAGTGCCCCTTCGGTGG

CCTCCAGATTCATTGATTCCCACACACCCCCTCTCCGCCCCATCCTGAAGAAGACGGCCA

GCCTGGGCTTCTGTGTCACCTACGTCTTCTTCATCACCAGCCTCATCTACCCCGCCATCT

GCACCAACATCGAGTCCCTCAACAAGGGTTCGGGCTCACTGTGGACCACCAAGTTTTTCA

TCCCCCTCACTACCTTCCTCCTGTACAACTTTGCTGACCTATGTGGCCGGCAGCTCACCG

CCTGGATCCAGGTGCCAGGGCCCAATAGCAAGGCGCTCCCAGGGTTCGTGCTCCTCCGGA

CCTGCCTCATCCCCCTCTTCGTGCTCTGTAACTACCAGCCCCGCGTCCACCTGAAGACTG

TGGTCTTCCAGTCCGATGTGTACCCCGCACTCCTCAGCTCCCTGCTGGGGCTCAGCAACG

GCTACCTCAGCACCCTGGCCCTCCTCTACGGGCCTAAGATTGTGCCCAGGGAGCTGGCTG

AGGCCACGGGAGTGGTGATGTCCTTTTATGTGTGCTTGGGCTTAACACTGGGCTCAGCCT

GCTCTACCCTCCTGGTGCACCTCATC

ATGG

GGGCCCGGAGCGGAGCTCGGGGCGCGCTGCTGCTG---GCACTGCTGCTCTGCTGGGACC

CGAGGCTGAGCCAAGCAGGCACTGATTCTGGCAGCGAGGTGCTCCCTGACTCCTTCCCGT

CAGCGCCAGCAGAGCCGCTGCCCTACTTCCTGCAGGAGCCACAGGACGCCTACATTGTGA

AGAACAAGCCTGTGGAGCTCCGCTGCCGCGCCTTCCCCGCCACACAGATCTACTTCAAGT

GCAACGGCGAGTGGGTCAGCCAGAACGACCACGTCACACAGGAAGGCCTGGATGAGGCCA

CCGGCCTGCGGGTGCGCGAGGTGCAGATCGAGGTGTCGCGGCAGCAGGTGGAGGAGCTCT

TTGGGCTGGAGGATTACTGGTGCCAGTGCGTGGCCTGGAGCTCCGCGGGCACCACCAAGA

GTCGCCGAGCCTACGTCCGCATCGCCTACCTGCGCAAGAACTTCGATCAGGAGCCTCTGG

GCAAGGAGGTGCCCCTGGACCATGAGGTTCTCCTGCAGTGCCGCCCGCCGGAGGGGGTGC

CTGTGGCCGAGGTGGAATGGCTCAAGAATGAGGATGTCATCGACCCCACCCAGGACACCA

ACTTCCTGCTCACCATCGACCACAACCTCATCATCCGCCAGGCCCGCCTGTCGGACACTG

CCAACTATACCTGCGTGGCCAAGAACATCGTGGCCAAACGCCGGAGCACCACTGCCACCG

TCATCGTCTACGTGAATGGCGGCTGGTCCAGCTGGGCAGAGTGGTCACCCTGCTCCAACC

GCTGTGGCCGAGGCTGGCAGAAGCGCACCCGGACCTGCACCAACCCCGCTCCACTCAACG

GAGGGGCCTTCTGCGAGGGCCAGGCATTCCAGAAGACCGCCTGCACCACCATCTGCCCAG

TCGATGGGGCGTGGACGGAGTGGAGCAAGTGGTCAGCCTGCAGCACTGAGTGTGCCCACT

GGCGTAGCCGCGAGTGCATGGCGCCCCCACCCCAGAACGGAGGCCGTGACTGCAGCGGGA

CGCTGCTCGACTCTAAGAACTGCACAGATGGGCTGTGCATGCAAAATAAGAAAACTCTAA

GCGACCCCAACAGCCACCTGCTGGAGGCCTCAGGGGATGCGGCGCTGTATGCGGGGCTCG

TGGTGGCCATCTTCGTGGTCGTGGCAATCCTCATGGCGGTGGGGGTGGTGGTGTACCGCC

GCAACTGCCGTGACTTCGACACAGACATCACTGACTCATCTGCTGCCCTGACTGGTGGTT

TCCACCCCGTCAACTTTAAGACGGCAAGGCCCAGCAACCCGCAGCTCCTACACCCCTCTG

TGCCTCCTGACCTGACAGCCAGCGCCGGCATCTACCGCGGACCCGTGTATGCCCTGCAGG

ACTCCACCGACAAAATCCCCATGACCAACTCTCCTCTGCTGGACCCCTTACCCAGCCTTA

AGGTCAAGGTCTACAGCTCCAGCACCACGGGCTCTGGGCCAGGCCTGGCAGACGGGGCTG

ACCTGCTGGGGGTCTTGCCGCCTGGCACATACCCTAGCGATTTCGCCCGGGACACCCACT

TCCTGCACCTGCGCAGCGCCAGCCTCGGTTCCCAGCAGCTCTTGGGCCTGCCCCGAGACC

CAGGGAGCAGCGTCAGCGGCACCTTTGGCTGCCTGGGTGGGAGGCTCAGCATCCCCGGCA

CAGGGGTCAGCTTGCTGGTGCCCAATGGAGCCATTCCCCAGGGCAAGTTCTACGAGATGT

ATCTACTCATCAACAAGGCAGAAAGTACCCTCCCGCTTTCAGAAGGGACCCAGACAGTAT

TGAGCCCCTCGGTGACCTGTGGACCCACAGGCCTCCTGCTGTGCCGCCCCGTCATCCTCA

CCATGCCCCACTGTGCCGAAGTCAGTGCCCGTGACTGGATCTTTCAGCTCAAGACCCAGG

CCCACCAGGGCCACTGGGAGGAGGTGGTGACCCTGGATGAGGAGACCCTGAACACACCCT

GCTACTGCCAGCTGGAGCCCAGGGCCTGTCACATCCTGCTGGACCAGCTGGGCACCTACG

TGTTCACGGGCGAGTCCTATTCCCGCTCAGCAGTCAAGCGGCTCCAGCTGGCCGTCTTCG

CCCCCGCCCTCTGCACCTCCCTGGAGTACAGCCTCCGGGTCTACTGCCTGGAGGACACGC

CTGTAGCACTGAAGGAGGTGCTGGAGCTGGAGCGGACTCTGGGCGGATACTTGGTGGAGG

AGCCGAAACCGCTAATGTTCAAGGACAGTTACCACAACCTGCGCCTCTCCCTCCATGACC

TCCCCCATGCCCATTGGAGGAGCAAGCTGCTGGCCAAATACCAGGAGATCCCCTTCTATC

ACATTTGGAGTGGCAGCCAGAAGGCCCTCCACTGCACTTTCACCCTGGAGAGGCACAGCT

TGGCCTCCACAGAGCTCACCTGCAAGATCTGCGTGCGGCAAGTGGAAGGGGAGGGCCAGA

TATTCCAGCTGCATACCACTCTGGCAGAGACACCTGCTGGCTCCCTGGACACTCTCTGCT

CTGCCCCTGGCAGCACTGTCACCACCCAGCTGGGACCTTATGCCTTCAAGATCCCACTGT

CCATCCGCCAGAAGATATGCAACAGCCTAGATGCCCCCAACTCACGGGGCAATGACTGGC

GGATGTTAGCACAGAAGCTCTCTATGGACCGGTACCTGAATTACTTTGCCACCAAAGCGA

GCCCCACGGGTGTGATCCTGGACCTCTGGGAAGCTCTGCAGCAGGACGATGGGGACCTCA

ACAGCCTGGCGAGTGCCTTGGAGGAGATGGGCAAGAGTGAGATGCTGGTGGCTGTGGCCA

CCGACGGGGACTGCTGA

>dog

CCCCAACCGCCGAGGCTGGCAAAGCGCACAGGCTAAGTGCTGAGGAGAGGGACCAGCTGC

TGCCAAACCTGAGGGCTGTGGGGTGGAACGAGGTGGAGGGCCGAGACGCCATCTTCAAGC

AGTTCCATTTCAAAGACTTCAATCGGGCTTTTGGCTTCATGACGAGGGTGGCCCTGCAGG

CTGAGAAACTGGACCACCATCCTGAATGGTTTAACGTGTACAACAAGGTCCACATCACCC

TGAGCACCCACGAGTGTGCCGGCCTTTCGGAACGGGACATAAACCTGGCCAGCTTCATCG

AACAAGTAGCGGTGTCCATGACCTAGGCCCCTGCCTTCCTCTCTGAACGCTTCAGGGGAA

G------GGGGTGACTGAACTGGGAACCCAGGGAGGGAATCGGGGAGCCCT

GTTTATGGTCTAGGT

TTAAAAAGAGATGTTTTAAGCTCATCAGGAAGATGCCTATTATTGGTCGCAAGATCCAAG

ATAAGGTGAACAAGACCAAGGATGATATTAGCAAGAACATGTCATTCCTGAAAGTGGACA

AAGAGTATGTGAAAGCTCTGCCCTCTCAAGGTCTGAGTGCATCTGCAGTTCTGGAGAAGC

TCAAGGAATACAGCTCTATGGACATCTTCTGGCAGGAGGGCAAAGCGTCTGGAGCAGTGT

ACAGCGGGGCGGAGGAGCTCACCGAACTCCTCGTGAAGGCTTATGGAGATTTTGCCTGGA

GCAATCCATTGCACCCAGATATCTTCCCAGGACTGCGCAAGATTGAGGCAGAGATCGTGA

GGATAGCTTGTTCCCTGTTTAATGGGGGGCCGGACTCCTGTGGGTGTGTGACCTCTGGGG

GGACAGAAAGCATACTGATGGCCTGCAAAGCTTACCGGGACCTGGCTTTTGAGAATGGGA

TCAAAACTCCAGAAATTGTGGCCCCCCAGAGTGCCCATGCTGCATTTGACAAAGCAGCCA

ATTATTTCGGGATGAAGATTATACGGGTTCCACTGAACAAAATGATGGAGGTGGATGTTC

GGGCAATGAGGAGAGCCATCTCCAGGAACACGGCCATGCTTGTCTGTTCTACCCCCCAGT

TTCCTCATGGTGTCATAGATCCCGTCCCTGAAGTGGCCAAGCTGGCTGTCCGATATAAAA

TACCTCTTCATGTAGATGCTTGTCTGGGGGGCTTCCTCATCGTCTTTATGGAGAAAGCAG

GATACCCACTGGAGCAACCATTTGATTTCCGGGTGAAAGGTGTGACCAGCATTTCAGCTG

ATACCCACAAGTATGGCTACGCCCCCAAAGGCTCTTCCGTGCTGTTATACAGTGACAAGA

AGTACAGGAGCCATCAGTTCTTCGTGGCGACTGACTGGCAGGGCGGCATCTACGCTTCCC

CGACCATCGCGGGCTCACGGCCCGGAGGCATTAGCGCGGCCTGTTGGGCTGCCTTGATGC

ACTTCGGTGAGAGCGGCTATGTTGAAGCCACCAAACAGATCATCAAAACTACTCGCTTCC

TCAAGTCAGAACTGGAAACTATCAAAGGCATCTTTGTTTTTGGGAATCCTCAGTTGTCAG

TAATTGCTCTGGGCTCCCGAGACTTTGACATCTACCGACTGTTCAACCTGATGACGGCTA

AGGGGTGGAACTTGAACCAGCTGCAGTTCCCGCCCAGTATTCATTTCTGCATCACGCTGG

TGCACACCCGGAAGCGAGTAGCCATACAGTTCTTAAAGGACATCCGGGAGTCGGTCACTC

AAATCATGAAGAATCCGAAAGCAAAGACCACAGGAATGGGTGCGATCTATGGCATGGCCC

AGACGACAGTTGACAGGAACCTGGTCGCAGAATTGTCCTCGGTCTTCTTGGACAGCCTCT

TCAGCACGGACACTGTGACTCCAAGCAGCCAGATGAATGGTTCTCCGAAACCCCGCTGA

ATGGCCATGGCCTCAGAGGATGACTACCGTCACAGTTCAAAT

TCCACCTACAGACCTGCAAGCAGCTCTCTCCGAGCTGACCAGGAAGCACTGCTCGAGAAG

CTGCTGGACCGCCCACCACCCAGCCTGCAGAGACCCAAGGACCGCTTCAATGGTGCCTAC

ATCATCTTCTTCAGCTTGGGCATTGGCGGCCTCTTGCCATGGAACTTCTTTATCACTGCC

CAAGAGTACTGGGTATTCAA--TTCAGAACTGCTC-AACCCAGTCGCAGGGGAGAACCC-

-ACAATTCAAAC-TTCTG-----------------------AACTACTTTGAGAGCTACC

TGACTGTTGCCTCCACCGTCTCCTCCGTGCTGTGCCTCATGGCGAACTTCCTGCTCGTCA

ACAGGGTTCCGATTCATGTCCGAGTGCTGGCTTCGCTGACCATCATGCTGGCCATCTTTT

TGGTGATGACCGTGCTGGTGAAGGTGGACACCTCCTCCTGGGCCTACGGCTTCTTTGCTG

TCACCATTGTCTGCATGGCGATCCTCAGTGGCACCTCCACCATCTTCAGTAGCAGTGTCT

TCGGCATGACTGGCTCCTTCCCCATGAGGAATGCCCAGGCGCTGATATCAGGAGGAGCCA

TGGGGGGCACCATCAGTGCCGTGGCCCTGCTGGTGGACCTGGCGGCGTCCAGCGACGTGA

CGGACAGCACCCTGGCCTTCTTCCTGACCGCAGACGTCTTCCTCGGGCTCTGCGTCGGAC

TCTACCTGCTGCTGCCGCGGCTGGAGTACGCCAGGTTCTACCTGAGGCCTGTTTGGCCGG

CCCATGTGTTTTCTGGGGAGGAGCAGCCGCCGCAGGACTCCCCCAGCGCCCCTCTGGCAG

CCCCTGGATCCAGCGAGTCCTCGACCCCACCCCTCTGGCCCATCCTGAAGAGGACAGCCG

GCCTGGGCTTCTGCATCCTCTACCTCTTCTTCATCACCAGCCTTGTCTTCCCTGCCATCT

CCACCAACATCGAGTCTGTTGACAAGGGCTCGGGCTCACTGTGGACCACCAAGTTCTTCG

TCCCCCTCACCACCTTCCTCCTGTTCAACTTTGCTGACCTGTGTGGCCGGCAGATCACAG

CCTGGATTCAGGTGCCAGGGCCCAGGAGTAAGGTCCTTCCCGGGCTTGTGCTCCTCCGGA

CCTGTCTCCTCCCCCTCTTCATGTTCTGCAACTACCAGCCCCGCATCCACCTGCACACGG

TGGTCTTCCAGTCCGACCTCTACCCGGTGCTTTTCACCTCATTGCTGGGACTTAGCAACG

GCTACCTCAGCACCCTGGCTCTTATGTACGGGCCGAAGATCGTGCCCCGGGAGCTGGCCG

AGGCCACGGGGGTGGTGATGTCCTTCTATGTGTGCTTGGGCTTGGTACTTGGCTCGGCCT

GCTCTGCCCTGCTCGTGCACCTCATC

ATGT

GGGCCCGGAGCGGAGCGCGGGGCGCGCTGCTGCTG---GCGCTGCTGCTCTGCTGGGACC

CGAGGCTGAGCCGAGCAGGCACTGATTCTGGCAGCGAGGTGCTCCCTGACTCCTTCCCGT

CGGCGCCGGCCGAGCCCCTGCCCCACTTCCTCCAGGAGCCCCAGGACGCCTACATCGTGA

AGAACAAGCCCGTGGAGCTGCGCTGCCGCGCCTTCCCTGCCACACAGATCTACTTCAAGT

GCAACGGCGAGTGGGTCAGCCAGAATGACCACATCACGCAGGAGGGCCTGGATGAGGCCA

CTGGCCTGCGGGTACGGGAGGTACAGATCGAGGTGTCACGGCAGCAGGTGGAGGAGCTGT

TCGGGCTGGAGGACTACTGGTGCCAGTGCGTGGCCTGGAGCTCCGCGGGCACCACCAAGA

GTCGCCGGGCCTATGTCCGCATCGCATACCTGCGCAAGAACTTCGATCAGGAGCCTCTGG

GCAAGGAAGTGCCCCTGGACCAGGAGGTTCTTCTGCAATGTCGCCCACCAGAGGGGGTGC

CTGTGGCTGAGGTGGAGTGGCTGAAGAACGAGGACATCATCGACCCCACCCAGGACACCA

ACTTCCTGCTCACCATCGACCACAACCTCATCATCCGCCAGGCCCGCCTGTCAGATACAG

CCAACTACACCTGTGTGGCCAAGAACATTGTCGCCAAGCGCCGAAGTACCACTGCCACGG

TCACTGTCTATGTGAACGGGGGCTGGTCCAGCTGGGCCGAGTGGACGCCCTGCTCCAACC

GCTGCGGCCGTGGCTGGCAAAAGCGCACCCGGACCTGCACCAACCCGGCCCCGCTCAACG

GAGGCGCCTTCTGTGAGGGCCAGGCCTTCCAAAAGACCGCCTGCACCACCGTGTGCCCAG

TCGATGGAGCGTGGACAGAGTGGAGCAAGTGGTCAGCCTGCAGCACCGAGTGTGCCCACT

GGCGCAGCCGCGAGTGCATGGCGCCTCCGCCCCAGAACGGAGGCCGAGACTGCAGCGGGA

CCCTGCTCGACTCCAAGAACTGCACGGACGGGCTGTGCGTGCAGAATAAGAAAACTCTAA

GTGACCCCAAAAGCCACCTTCTGGAGGCCTCGGGGGATGTGGCTCTGTACGCGGGCCTCG

TGGTGTCCATCTTCGTCGTCGTGGGCGTCCTCATGGTGGTGGGGGTGGTGGTGTACCGCC

GCAACTGCCGGGACTTCGACACGGACATCACCGACTCATCCGCTGCCCTCACTGGCGGCT

TCCACCCCGTCAACTTCAAGACTGCGAGGCCCAACAACCCACAGCTCCTGCACCCGTCTG

TGCCTCCGGACCTCACGGCCAGTGCTGGCATCTACCGCGGGCCCGTGTATGCCCTGCAGG

ACTCTGCGGACAAGATCCCCATGACCAACTCCCCCCTGCTGGACCCCTTGCCCAGTCTCA

AGATCAAGGTCTACAACTCCGGCACCACCGGCTCTGGGCCAGGCCTGCCAGATGGGGCCG

ACCTGCTGGGGGTTGTGCCGCCTGGCACGTACTCTGGCGATTTCACCCGGGATGCCCACT

TGCTGCACCTGCGCAGTGCCAGCCTCGGCTCCCAGCAGCTCCTGGGCCTGCCCCGTGACC

CGGGGAGCAGCGTCAGCGGCACCTTTGGCTGCCTGGGTGGGAGACTCAGCATCCCCGGCA

CAGGGGTCAGCCTGCTGGTGCCCAATGGAGCCATCCCCCAGGGCAAGTTCTATGAGATGT

ACCTCCTTATCAACAAAGCAGAAAACACCCTCCCGCTTTCAGAAGGGACCCAGACAGTCC

TGAGCCCCTCGGTGACCTGTGGGCCCACGGGCCTCCTGCTGTGCCGCCCCGTCATCCTCA

CAGTACCCCACTGTGCTGAAGTCAGCGCCGGCGACTGGATCTTCCAGCTCAAGACCCAGG

CCCACCAGGGCCACTGGGAGGAGGTGGTGACTCTGGACGAGGAAACCCTGAACACCCCCT

GCTACTGCCAGCTAGAGGCCAGGTCGTGCCACATCCTGTTGGACCAGCTGGGCACCTACG

TGTTCACGGGTGAATCCTATTCCCGCTCGGCAGTGAAGCGGCTCCAGCTGGCCATCTTCG

CCCCTGCCCTCTGCACCTCCCTGGAGTACAGCCTCAGAGTCTACTGCCTGGAGGACACCC

CTGTGGCCCTGAAGGAGGTGCTAGAGCTGGAGCGGACCCTGGGAGGCTATCTAGTGGAGG

AGCCCAAACCCCTGCTGTTTAAAGACAGTTACCACAACCTGCGCCTCTCTCTACATGACA

TCCCCCACGCCCACTGGAGAAGCAAGCTGCTGGCCAAGTATCAGGAGATCCCCTTCTATC

ACATCTGGAGCGGCAGCCAGAAGGCCCTGCACTGTACTTTCACCCTGGAGAGGCACAGCC

TGGCCTCCACAAAGCTCACCTGCAAGATCTGTGTGCGGCAGGTGGAAGGGGAGGGCCAGA

TCTTCCAGCTGCACACCACTCTGGCAGAGACGCCTGCCGGCTCCCTGGACACCTTCTGCT

CTGCCCCTGGCAGCACAGTCACCACCCAGCTGGGACCCTATGCCTTCAAGATTCCACTGT

CCATCCGCCAGAAGATATGCAACAGCCTGGATGCCCCTAACTCACGGGGCAATGACTGGC

GGCTCTTGGCGCAGAAGCTCTCCATGGACCGGTACCTGAACTACTTTGCCACCAAAGCGA

GCCCCACGGGTGTCATCCTGGACCTCTGGGAAGCTCTGCAGCAGGACGACGGGGACCTCA

ACAGCCTGGCGAGTGCCTTGGAGGACATGGGCAAGAGTGAGATGCTGGTGGCCATGGCCA

CTGACGGGGACTGCTGA

Alignments of tandem elements

>mouse

GGCTTTGCCATTTTACCAGTGGGCCTTTGTTGGCTGAAAGAGACCTT-TAGATATTCCAA

GCAGTCAGATGCAACAGTGGGCCGAGGCTATTGCATTCAATCGGGCAGGTTGTTTACTGA

ACAGGGAACAGTGCTTTTGGCGGAGGAGCTGCTAGGAAATGAATCCAGCCTCACTGAACT

GCCAACCCCGGGCTTTCCAGGATGGGACTGAATCTGCCCAGAACATAAAGACATCGTGTG

GACCAGTGGTGGGTGCCTTGAGAGGGCTTTCCTATAGGGCTGCTGCCAGCCTCAGTTTCC

TGCTT-----CATA---ATAATCC-CTTTCCTCAAATG----TATCCCTCAAGGGACATG

GGACCAGTGAGCGGCAGAGAAAAATCGATAGACTACAAGCGTGCGTGGAGTGCCTGGAAG

ATAAGCTTTGGGACAA---GGGGAGCA-----------GAGTGCTTTCTTCATAATAGCA

GTGGGGAT--GGAGAGGGCTCAGGGTGTCTAGTCGGGAGGG--AGGGATGTGTGTGACAG

CCACT----TGTGGACCTTGGCGTTTAAATCTGTCCCCTCTTGCTGGCCTGATTTCTCAG

GTCACCCAGTGCCT-GCCCTGGCGATT------GGCCCTCACGGCCTTCAATGATTCCT-

----------------GTTCATCTCC-----------CT---------------------

--CCACCGGATCAATCACAGACCCACAAACCC-----TTCTTCAGTGCAAAGCC------

----TGTGAATGTGCA-GCTTAGGT-CCAAGCCTGGCTCCATCCCCAC-ACTTGTC----

----TCTGCTCAGCATCCCT-TCCAGACTTCCATTTCC---------AAGCATGGTCCTG

CCTGTGCTGCCGGGGCCAGCCCGCCCCTCTGGATGGTGCAGCCTGGAGTCATACACAAGC

ACCTG----CAGATTCCCCTAC----CCCCCACCTCCTCTCTGAAGG-AGCCCAGATAAA

ACAGAG-CTAATCCACCCC-GGGAGAAGGGCTCTGTGCCTGCGGGGACCCAGGCACAAAT

TGGGATGTCCTGCCGCATTACTTCTGCTGAGCTGGACATCAACATCTCACCTCC-GCACC

CAGGCAAGCCGGGGACAGCCAAGGGAGTGGAAAGGCGGTGGGGGAAGGGA--CCAAGCAG

CCACCAGGCCTCC----------ATCAAAGGCGAT-GGAGAGAGAGCAG-CCCAGCCAGA

GAGGATTTTTAATGACCCCAAATCCTGGCACCTCATTATGACTCTGATAAGGTGATGGG-

GCTGTCACTTCCAGATGCAGCCAAGG--GCTCCCCAGAGAAATTTTCCTGCCTTCCTGCT

AATCTTCTTATAAACATTGTTATTTATTTTCCCTTATTTTATCCGACATGTGTCAAGCAG

CCCCTGCTAAGCCTGTGACATCTCCAGGGAGACCAGAGGAGGTTGG---------CCTGT

CACTCCCCCAGTCCTGCCCC-CT--------------TCCCAGGCCCTATTCTCTAGCCC

AACACATGG--GGGCCTGGACGGATAATTTTCCGATGTCTCTAAGAGTTTGGACT-GGGC

CAGA----ACTGGGCAACTGGGATCTAGGTGTTGA-GTTAGCAGGTCACAGGCCATGCCA

ACCGGGGCTGGTTCCAAAGGAAA-

>rat

GGCTTTGCCATTTTACCAGTGGGCCTTTATCGGCTGAAAGAGAC-TT-TGGATATTCCAA

GCAGTCAGATGTAAGAGAAGG-----GATATTCCATTCGATCGGACAGGTTGTTTACTGA

ACAGAG----GTGCTT--GGCTGGGGAACAACCAGGAAATGAATCCAGCCTCACTGTACT

ATCAACCCTGGGTTTTCCAGAATAGGACTGAATCTGCCCAGAACATGATGACACCCTGTG

GACCAGTGGT----GCCTCGAGAGGGCTTTCCTATAGGGCTGCTGTCAGCCTCAGTCTCT

TA---------ATA---ATGCTCCTCCTTGCTCAAACC----TATCCCTCAAGGGACATG

GGACCAGTGAGCGGCTGAGAGAA-TCTATAGACCACAATCGTGCTTGGAGTGCCAGGAAG

ATAAGCTTTGGGACAA---GGGACGTG-----------GAGTGCTTCCTCCATAATACCA

GTGGGGAT--GGAGAGGGCTCAGGGTGTCTAGTCGGGAGGG--AGGAATCTGTGTGACAG

ACAAC----TA-----CTTGGC-TTTGAATCTGTCCCCTCTTGTTGGCCTGACTTCTCAG

GTCACCCAGTGTCT-GCCCTGGCGATT------GGTCCTCACTGCCTTCAATGGTTCCAA

CAGACATGGAAAAATGGTTCCTGCTCT---------GCT---------------------

--CCCCCCACCCGATCACAGACCCACAAAGCC-----TTC---AATGCAGGGC-------

----TGTGAATGCACA-GCTCGGGT-CAAGGACTGGCTCCATCCATAC-ACTTGTCAATC

AATCCCTGCCCAGGATCCCT-TCCAGACCTCGGTTACT---------AAGCGTGGTCTTG

CCTGGGCAGCAGGG-----CCAGCCCCTCTGGA--GTGCAGCCTGGAGTCATACACATGC

GCCTG----CAGGTCCCCCTAC----CCCC-ACTTCATCTCTGAAGG-AGCCCAGATAAA

ACAGAG-CTAATCCACACA-GGGAGAAGGGCTCTGTGCCTGCGGGGACCCAGGCACAAAT

TGGGATGTCCTGCTGCATTACTTCCGCTGAGCTGGGCATCAACCTCCCACCTCCAGCACC

CAGGCAAGT-GGGGATAGTCAAGGGGGTGGGATGGCGGTGGGGGAAGGGA--CCAAGCAG

CCACCAGGCCTCC----------ATCAAAGGCGAT-GGAGAGAGGGCAG-CCCAGC-AGA

GAGGATTTTTAATGACCCCAAATCCTGGTACCTCATTATGACTCTGATAAGGTGATGGG-

GCTGTCACTTCCAGATGCAGCCAAGG--GCTCCCTGGAGAAATTTTCCTGCATTCCTGCT

AATCTTCTTATAAACATTGTTATTTATTTTCCCTTATTTTATCCGACATGTGTCAAGCAG

CCCCTGCTAAGCCTGTGACATCTCCAGGGAGACCAGAGGAGGTTGG---------CCTGT

CAGTGCCATAGTCCTGCCCC-CT--------------TTCCAGGCC-TATTCTCTAGCC-

GACATGTTGTCGGGCCTGAATGGGTAATTTTCTGATGTCTCTAAGAGTTTGGGCT-GGGC

CAGA----ACTGGGCAACTGGGATCCGGGTGTTAA-GTTAGCAGGTCGCAGGCCATGTCA

ACTGGGGCTGGTTCCAAAGGAAA-

>human

-CTTTTACCATTTTACCAGAGGCCCTCGTCTGGCTGGGAGAGACCTCAGGACCAACCTGG

GCAGTTAGTTTCGACAGCAAG-----CCTGCCACATACAGTTGGGCAGGTTGTTAACTGC

ACAAAGCTGCTTGTCT--GAGGGGGTGATTATGGGGGCTGGAATCCAGCTTCACTCTTGT

CGCCAAGCCACACTCCCCAGTGTGGGACA--ATCTACCGAGAGCACAAAGGTACCATGTG

GGCCAGTGGT----GCCATAGGGGGCCAC-CCAGTGGAGCTGCCCCCAGCCTCAGCTTGC

TGAATTGAAACACAAGGGTAATCCTCTCTCCTCCAAACTCCTCATCCCTCAAGGGAAGTG

GGGCCAGTGAGTGGTTGAGAAAA-TCAATACACTACAA--GTGCTTTGAGCTCCTTGAAG

ATAAGCTTTGTGACCAAGAGGGAGGTGATTAAAGGCAGGAGCTCTTTCTCTGTGGCACTG

ATAGGGATGGAGGAACGGGTCAGGCTGTCTGGGATGGGGATTTGCATGTGTGCATGGCAG

ATACTGGCCTCTGGACACTGGCGTTAGCATCCATCCC-TCCTGCTGGACTGGGGGCTGAA

GCTTGTGTCTCCCTTGTTTCTGAGCCCCTCCATGTCTCTCCCCGTGACTTGCACTTTGCA

GAAGATCAATACACA--TTTGTTCTCGCTGAATTCAATTTTCTGTACATCCTATCATCGC

GCTCCTCCATTCTGCTCCCTGTACAAACATGCAGTCATTCATGAGCACACACCCAACCTC

GGGCTGGGAATCCCGAGACCTGTGTCCAAAGCCTGACTCTGCCCCCACGATGCTGCAACC

----TATGTACACTGCCTCTGCCCGAACCTCAGTTTCCCCATGTGCCAAGCGAGGCATTG

GATAATATGGGTGGC---AGGCTCCTTACCGCACAACGCAGGCTGGAGTCGCGGATGCCT

CATGG---CTATGCGCCTATGCAGAGCCCCCCTTTTGTCTCTGAAGG-AGCGGAGATAAA

ACGGAGCCTAATCCGCACCAGGGAGACGAGTTCTCTGCCCGAGGGGCCAAGGGCACAAAT

TGGGCCGTCGTGTTGCATTACTTCCGCCAAGCTGGGCTCCACCATCTCACCTCTGTACCC

CTGCAGACA--GGGACAG-TGATGGGCAGACAGCGCAGTGGGGGCAGGGGACCTGAGCCA

CCACGAGGCCCCCTGTCTCCACCATCAAAGGCAATGGGCGAGGGAGGGGGCCCAGCCCAG

GAGGATTTTTAATGACCCCAAATCCTGGCAACTCATTATGTCTCTGATAAGGCAATGGGA

GCTGTCATTCTGAGATGCAGCCAAGGAGGCTCCCTAGAGAAATTTTGCCGCCTTCTCGCT

AATCTTCTTATAAACATTGTTATTTATTTTTTCTTATTTTATCCGACATGTGTCAAGCAG

CCCCTGCTAAACCTGTGACATCTCCAGGGAGGCTGGCAGAGGTTTGGGGTTGG--CCTGT

C-CCTCCCCAGCCCTCTCCAGCCCTGGCGCACAGCCCCTATAGGCC-TGTTCTCTGGGCA

GCCCACGTT--CTGTCCACGCTGGCAGCTTTGAGATGCCCACAGGAGCTGGGTCTTGGGC

CAGGAGACACAGGCCATCTGGGAGCTGGCCGTCAA-GTGAGTGGATCACAGACTATGCCA

GTTGGGGCTGGTTCCAAGGAGAAG

>dog

-GCTTTACTATTTTGCCAGAGA------TCTAGTTGAGAGAGACCTCATGGCCATCCTCT

GCTGTTAAATGTGATAGCAGG-----GCTGCCACACACAGTTGGGCAGGTTGTTCACTG-

----------------------------------------GGATCCAGCCTCACTCTGCC

CACCAAGGCACCTCTTTCAGCATGTGGCTGTATTTGCCCAGGGCACAAAGGTACCATACG

GGCCAGTAGC----ACCATGAGTGG-------------GCCGCTCCCAGCCTCAGTTTCC

TGAACTGAGACACA-GGGTAATCCTTCCTCTTTCAAGC-CTCCATCCCTCAAGGGAAGCG

GAGCCCGTGAGTGGTTGAGAAAA-TCAATACACTACAA--GTGCTTTGAGCTCCTTGAAG

ATAAGCTTC-TGACAAAGAGGGAGGTGATGGAAGGCGGGAGCCCTTTCCCGGGAGC-CTG

ACAGGGGCTTGGGAATGGATCAGAAAGTCTGGG-----------TGAGCGTGTGGGACAG

ATGCTGGCCTGTGGACACCGGCTTCTGCATCCATCCT-TCCTGCCCGCCTGGGGCCGCAG

ACTCGTGTGTCCCT-GCGTCTGGGGTCC-CTGCGTCTTGCCCAGCCACCTGCACGTTGTG

GGAGACAGGGGCGCAGTTTTTTCCTCATGAAATTGAATT---------------------

--TCCTCCGCTCTGTTCCCTATGCACGGATGCAGTAGCCCCCTAA-ACACACCCAGCTTC

---CTGGGAGCCTGGACGCCTGGGTTCAAAGCCTCACTCTGCCCCCGATACGCAGCCACC

----TCTGACCTCTGCCCATCCCCGGACTTGAGTTTCA---------AAGTGGGGTGCTA

GA-----CGGCCGGC---GGGGCTCCCTCCCTACAGGGCAGTCTGGAGCTGCAGAGGCCT

CGTCGTGTCCACGTGTCCACACAGAGCCCCTCTTTTGTCTCTGAAGGCAGCTGAGATAAA

ACAGAGTCTAATCCGCACCAGGGAGACGGGTCCTCTGCCCGAGGGGCCGCGGGCACAAAT

TGGGCCGTCGTGTTGCATTACTTCCGCCAAGCTGGGCCCCACCATCGCACCTCTGTCCCC

CCAGCGAAT--GGGACAG-CGAGGGGCAGACAGCACAGTGGGGGCAGGGGGCCCGGGCCG

CCACCAGGCCCCCTGACTCCACCATCAAAGGCGACAGGAGAGGGAGGGGGCCCAGCCGCG

GAGGATTTTTAATGACCCCAAATCCTGGCAACTCATTACGTCTCTGATAAGGCAATGGGA

GCTGTCACTCCCAGATGCAGCCAAGGAGACTCCCTAGAGAAATTTTGCCGCCTTCTCGCT

AATCTTCTTATAAACATTGTTATTTATTTTTCCTTATTTTATCCGACATGTGTCAAGCAG

CCCCTGCTAAGCCTGTGACATCTCCAGGGAGGCTGGAGGAGGCTGGGGGGAGGGCCCTGT

C-CCCTCCCAACCCTCCCCAGCT--------CGGCCCTTGCCGCCC-TGCTCTCTTGCTG

GCCAAGCTT--CTAACCCAGCCGGTGGCTCCGCGATGCCCGCAGGAGCTGGGTCTTGGGC

CAGGCGACACTAGACATCTGGGGACTGGTCGTCAGTGCGGGGGGACTGCAGGCTGTGCTG

ACTGGGGCTGCTTCCAAGGAGAA-
